# Temporal enrichment of comammox *Nitrospira* and *Ca.* Nitrosocosmicus in a coastal plastisphere

**DOI:** 10.1093/ismejo/wrae186

**Published:** 2024-10-07

**Authors:** Qian Yang, Yin Zhong, Shi-wei Feng, Ping Wen, Heli Wang, Junhong Wu, Sen Yang, Jie-Liang Liang, Dan Li, Qiong Yang, Nora F Y Tam, Ping’an Peng

**Affiliations:** State Key Laboratory of Organic Geochemistry and Guangdong-Hong Kong-Maco Joint Laboratory for Environmental Pollution and Control, Guangzhou Institute of Geochemistry, Chinese Academy of Sciences, No. 511, Kehua Street, Tianhe District, Guangzhou 510640, P. R. China; Guangdong Key Laboratory of Environmental Protection and Resources and Utilization, No. 511, Kehua Street, Tianhe District, Guangzhou 510640, P. R. China; College of Resources and Environment, University of Chinese Academy of Sciences, No. 1, Yanqihu East Road, Huairou District, Beijing 100049, P. R. China; State Key Laboratory of Organic Geochemistry and Guangdong-Hong Kong-Maco Joint Laboratory for Environmental Pollution and Control, Guangzhou Institute of Geochemistry, Chinese Academy of Sciences, No. 511, Kehua Street, Tianhe District, Guangzhou 510640, P. R. China; Guangdong Key Laboratory of Environmental Protection and Resources and Utilization, No. 511, Kehua Street, Tianhe District, Guangzhou 510640, P. R. China; Institute of Ecological Science, Guangzhou Key Laboratory of Subtropical Biodiversity and Biomonitoring, Guangdong Provincial Key Laboratory of Biotechnology for Plant Development, School of Life Sciences, South China Normal University, No. 55, Zhongshan Dadao Xi Road, Tianhe District, Guangzhou 510631, P. R. China; Institute of Ecological Science, Guangzhou Key Laboratory of Subtropical Biodiversity and Biomonitoring, Guangdong Provincial Key Laboratory of Biotechnology for Plant Development, School of Life Sciences, South China Normal University, No. 55, Zhongshan Dadao Xi Road, Tianhe District, Guangzhou 510631, P. R. China; State Key Laboratory of Organic Geochemistry and Guangdong-Hong Kong-Maco Joint Laboratory for Environmental Pollution and Control, Guangzhou Institute of Geochemistry, Chinese Academy of Sciences, No. 511, Kehua Street, Tianhe District, Guangzhou 510640, P. R. China; Guangdong Key Laboratory of Environmental Protection and Resources and Utilization, No. 511, Kehua Street, Tianhe District, Guangzhou 510640, P. R. China; College of Resources and Environment, University of Chinese Academy of Sciences, No. 1, Yanqihu East Road, Huairou District, Beijing 100049, P. R. China; State Key Laboratory of Organic Geochemistry and Guangdong-Hong Kong-Maco Joint Laboratory for Environmental Pollution and Control, Guangzhou Institute of Geochemistry, Chinese Academy of Sciences, No. 511, Kehua Street, Tianhe District, Guangzhou 510640, P. R. China; Guangdong Key Laboratory of Environmental Protection and Resources and Utilization, No. 511, Kehua Street, Tianhe District, Guangzhou 510640, P. R. China; College of Resources and Environment, University of Chinese Academy of Sciences, No. 1, Yanqihu East Road, Huairou District, Beijing 100049, P. R. China; State Key Laboratory of Organic Geochemistry and Guangdong-Hong Kong-Maco Joint Laboratory for Environmental Pollution and Control, Guangzhou Institute of Geochemistry, Chinese Academy of Sciences, No. 511, Kehua Street, Tianhe District, Guangzhou 510640, P. R. China; Guangdong Key Laboratory of Environmental Protection and Resources and Utilization, No. 511, Kehua Street, Tianhe District, Guangzhou 510640, P. R. China; College of Resources and Environment, University of Chinese Academy of Sciences, No. 1, Yanqihu East Road, Huairou District, Beijing 100049, P. R. China; Institute of Ecological Science, Guangzhou Key Laboratory of Subtropical Biodiversity and Biomonitoring, Guangdong Provincial Key Laboratory of Biotechnology for Plant Development, School of Life Sciences, South China Normal University, No. 55, Zhongshan Dadao Xi Road, Tianhe District, Guangzhou 510631, P. R. China; School of Environment and Civil Engineering, Dongguan University of Technology, No. 1, Daxue Road, Songshanhu District, Dongguan 523808, P. R. China; Guangdong Neilingding Futian National Nature Reserve, No. 1, Mangrove Road, Futian District, Shenzhen 518040, P. R. China; School of Science and Technology, Hong Kong Metropolitan University, 30 Good Shepherd Street, Ho Man Tin, Kowloon, Hong Kong 999077, P. R. China; State Key Laboratory of Marine Pollution and Department of Chemistry, City University of Hong Kong, 83 Tat Chee Avenue, Kowloon, Hong Kong 999077, P. R. China; State Key Laboratory of Organic Geochemistry and Guangdong-Hong Kong-Maco Joint Laboratory for Environmental Pollution and Control, Guangzhou Institute of Geochemistry, Chinese Academy of Sciences, No. 511, Kehua Street, Tianhe District, Guangzhou 510640, P. R. China; Guangdong Key Laboratory of Environmental Protection and Resources and Utilization, No. 511, Kehua Street, Tianhe District, Guangzhou 510640, P. R. China

**Keywords:** Comammox nitrospira, coastal plastisphere, N_2_O, nitrogen cycling, mangrove

## Abstract

Plastic marine debris is known to harbor a unique microbiome (termed the “plastisphere”) that can be important in marine biogeochemical cycles. However, the temporal dynamics in the plastisphere and their implications for marine biogeochemistry remain poorly understood. Here, we characterized the temporal dynamics of nitrifying communities in the plastisphere of plastic ropes exposed to a mangrove intertidal zone. The 39-month colonization experiment revealed that the relative abundances of *Nitrospira* and *Candidatus* Nitrosocosmicus representatives increased over time according to 16S rRNA gene amplicon sequencing analysis. The relative abundances of *amoA* genes in metagenomes implied that comammox *Nitrospira* were the dominant ammonia oxidizers in the plastisphere, and their dominance increased over time. The relative abundances of two metagenome-assembled genomes of comammox *Nitrospira* also increased with time and positively correlated with extracellular polymeric substances content of the plastisphere but negatively correlated with NH_4_^+^ concentration in seawater, indicating the long-term succession of these two parameters significantly influenced the ammonia-oxidizing community in the coastal plastisphere. At the end of the colonization experiment, the plastisphere exhibited high nitrification activity, leading to the release of N_2_O (2.52 ng N_2_O N g^−1^) in a 3-day nitrification experiment. The predicted relative contribution of comammox *Nitrospira* to N_2_O production (17.9%) was higher than that of ammonia-oxidizing bacteria (4.8%) but lower than that of ammonia-oxidizing archaea (21.4%). These results provide evidence that from a long-term perspective, some coastal plastispheres will become dominated by comammox *Nitrospira* and thereby act as hotspots of ammonia oxidation and N_2_O production.

## Graphical Abstract

**Graphical Abstract:**
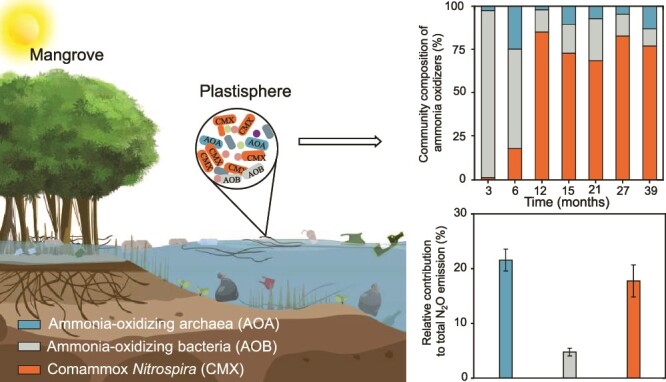

## Introduction

Plastic debris has emerged as a major and growing marine issue in recent decades. Approximately 82–358 trillion plastic particles weighing 1.1–4.9 million tonnes entered the oceans in 2019 [1], and at least 77% of positively buoyant marine plastic debris was beached or floated in coastal waters [2]. Microorganisms rapidly colonize on the surface of plastic debris and form biofilms with diverse microorganisms embedded in a matrix composed of extracellular polymeric substances (EPS), commonly called “plastisphere” [3, 4]. The formed biofilm can be a vector for the transportation of harmful algal species, potential pathogenic microorganisms, antibiotic resistance genes, and persistent organic pollutants, posing a potential threat to human and wildlife health [3–5]. In coastal environments, the plastisphere has been frequently reported to alter the biogeochemical recycling of elements [6–9].

The formation process of the plastisphere includes the attachment of microorganisms on the surface of plastics, the secretion of EPS, and the proliferation of microorganisms [5]. EPS can play an important role in biofilm formation by aggregating cells, facilitating interactions between cells, and providing nutrients for biofilm communities [10]. Polysaccharides (PS) and proteins (PN) are the main components of EPS, which can modify the hydrophobicity of the plastic surface and influence community development in the plastisphere [10, 11]. Different compositions of EPS have distinct impacts on the aggregation of microorganisms involved in nitrogen cycling and nitrogen removal in biofilm systems [12, 13]. Moreover, plastics always persist in coastal environments for a long period of time, and the composition of EPS on the surface of polyethylene (PE) exposed to coastal water has been found to change significantly over time [14]. It is therefore important to explore the temporal dynamics of nitrogen-cycling microorganisms in the coastal plastisphere over long time scales (at least years) and the associated effects on nitrogen biogeochemical cycling in coastal ecosystems. However, this area has seldom been researched and there is limited reported information.

Coastal environments are heavily impacted by anthropogenic inputs of ammonium nitrogen [15], which can be eliminated by nitrification, a fundamental component of the global nitrogen cycle [16] with the generation of a greenhouse gas nitrous oxide (N_2_O) [17]. Microbial-mediated nitrification was thought to involve two steps. First, ammonia is oxidized to nitrite by ammonia-oxidizing archaea or bacteria (AOA or AOB), and nitrite is then oxidized to nitrate by nitrite-oxidizing bacteria (NOB). In 2015, this notion was revolutionized with the discovery of comammox *Nitrospira* (CMX), which can perform the complete process of ammonia oxidation to nitrate [18, 19] (Fig. S1). CMX possess all genes encoding enzymes required for complete nitrification, including *amo* genes encoding sub-units of ammonia monooxygenase, *hao* genes encoding hydroxylamine dehydrogenase, and *nxr* genes encoding sub-units of nitrite oxidoreductase [18, 19]. All currently known comammox microorganisms belong to the genus *Nitrospira* and have been reported to be widely distributed in natural and engineered ecosystems [20–22]. CMX can generate more energy per molecule of ammonia than AOA and AOB, allowing CMX to be more competitive and become the dominant ammonia oxidizer in oligotrophic environments [18, 19, 23, 24]. Recent studies have revealed the high abundance and diversity of CMX in coastal wetland sediments and their contribution to the production of N_2_O [21, 25, 26]. However, the distribution and function of CMX in the coastal plastisphere have not yet been explored. Differences in the ecological niche between AOA, AOB, and CMX in the coastal plastisphere, and their contributions to nitrogen cycling and N_2_O production in the coastal ecosystems, remain unknown [27].

This study aimed to profile the long-term dynamic changes in the microbial community and function of the plastisphere in coastal ecosystems using a 39-month field colonization experiment with plastic ropes placed in the intertidal zone of a mangrove wetland. The temporal dynamics of ammonia oxidizers of the plastisphere were characterized together with factors potentially influencing activity using a combination of EPS analysis, 16S rRNA gene amplicon sequencing, metagenomic analysis, and quantitative PCR (qPCR). At the end of the colonization experiment, we also conducted a 3-day laboratory nitrification experiment to investigate the potential roles of AOA, AOB, and CMX in ammonia oxidation and N_2_O production in 39-month-old plastisphere and sediment samples. Results from this study provide insights into the temporal dynamics of plastic-associated nitrifying microorganisms and their crucial roles in nitrogen biogeochemical cycling and N_2_O emissions in coastal environments with severe plastic pollution.

## Materials and methods

### Colonization experiment

Discarded plastic ropes were chosen as the representative plastic in this study, as they are extensively distributed in coastal environments due to human activities, including shipping, fishing, aquaculture, and tourism [28]. Consumer plastic ropes were purchased from Hongxing Plasticizing Co., Ltd. (Guangdong, China), and the ropes consisted of PE, polypropylene (PP), and polyethylene terephthalate (PET) (Fig. S2). Plastic ropes were fixed to a bamboo raft located in the intertidal zone (foreshore position) of Futian Mangrove (22°52′ E, 114°02’ N), Shenzhen Bay, South China (Fig. S3A). This site is highly urbanized, characterized by a dense population and a well-developed economy. A total of 80 plastic ropes (1 cm width × 50 cm length) were tied to a bamboo raft (6 m × 5 m), which was attached to two stone pillars with hemp ropes and floated in the intertidal zone for 39 months from 2019 to 2022 (Fig. S3B). The plastisphere (also termed biofilm on the surface of plastic) and sediments (surrounding the plastic ropes) were collected at 3, 6, 12, 15, 21, 27, and 39 months. The plastisphere and sediment samples were scraped off with a sterile stainless steel spoon. At each sampling time, 3 g plastisphere was retrieved from the site with three replicates, and 5 g fresh surface sediments (0–5 cm) were obtained with three replicates. All samples were stored in sterile sampling bags or bottles at 4°C and transported to the laboratory immediately. These samples were used for EPS analysis, DNA extraction for 16S rRNA gene amplicon sequencing, and metagenome sequencing. Concentrations of carbon and nitrogen in the plastisphere or sediments were analyzed using an elemental analyzer (EA-IsoLink, Thermo Scientific, USA) [8] with 5 mg freeze-dried samples. Seawater samples at a depth of 10–20 cm were collected using a water collector at 3, 6, 12, 15, 21, 27, and 39 months. After filtration with a 0.22 μm disposable filter membrane (Jingteng, Beijing, China), physicochemical parameters were determined including pH, dissolved oxygen, temperature, salinity, NH_4_^+^, NO_3_^−^, and NO_2_^−^, as described previously [8].

### EPS extraction and analysis

EPS obtained from plastisphere and sediment samples were analyzed using the established Dowex-resin method [29]. Briefly, 20 mL of artificial seawater was added to 2 g of fresh plastisphere or sediments, with the addition of 0.1 g activated Dowex 50 W × 8 (hydrogen form, 200–400 mesh, Sigma–Aldrich, MO, USA). The mixture was continuously agitated in the dark at 4°C for one hour and centrifuged for 10 min at 4°C (4000 × *g*). The supernatant was collected and mixed gently with −20°C ethanol to attain a final concentration of 75% (v/v), stored overnight at −20°C, and then centrifuged for 15 min at 4°C (4000 × *g*). EPS in the supernatant was recovered and freeze-dried for subsequent analysis.

The concentration of PS was determined using the phenol sulfuric acid assay, while PN was measured based on the modified bicinchoninic acid (BCA) protein assay kit (Sangon Biotech, Shanghai, China). Three-dimensional excitation-emission matrix (EEM) spectra of EPS were recorded by a fluorescence spectrometer (Hitachi F-7000, Japan) using the scan mode with a 700-voltage xenon lamp at normal room temperature.

### Nitrification microcosm experiment

At the end of the colonization experiment, the 39-month-old plastisphere and surrounding sediments were collected, as described above. The nitrification microcosm experiment was conducted according to methods described previously [30–32], consisting of four treatments with different ammonia oxidizer inhibitors to distinguish nitrification activity and N_2_O production resulting from AOA, AOB, and CMX in ammonia oxidation (Fig. S4). Treatment I: control without any addition of inhibitors; treatment II: addition of 0.03% (v/v) 1-octyne to inhibit AOB; treatment III: addition of DMPP (3, 4-Dimethylpyrazole phosphate) to inhibit both AOB and CMX; and treatment IV: addition of 0.01% (v/v) acetylene to fully inhibit ammonia-oxidizing microorganisms (AOA, AOB, and CMX). In each treatment, 2 g of 39-month-old plastisphere or sediment samples were added into 50 mL serum bottles with or without inhibitor.

Each serum bottle was sealed with a butyl rubber stopper and aluminum cap and incubated at 25°C in the dark for three days. Triplicate serum bottles from each treatment were destructively sampled at 0, 24, 48, or 72 h. In each bottle, 50 ml gas was collected using a gas-tight needle (Agilent, California, USA) for the analysis of N_2_O by a Shimadzu QP2010 plus GC–MS (Kyoto, Japan). The analysis was performed using a GS-Carbon PLOT (30 m × 32 mm × 3 μm) chromatographic column in scan mode with a helium flow rate of 1.5 mL min^−1^ at the inlet. The temperature of the injection port was set at 160°C, and the GC oven was programmed to hold at 35°C for 3 min. The NH_4_^+^ in the plastisphere and sediments were extracted with 2 M KCl (1:5, m/v) and analyzed using the indophenol method [33]. The N_2_O yields of ammonia-oxidizers were determined as the amount of N_2_O-N (ng) produced per NH_4_^+^-N (μg) oxidized [27]. Nitrification and N_2_O production rates were calculated as described in Supplementary Text (Text S1).

### 16S rRNA gene amplicon sequencing

Forty-nine genomic DNA samples from the plastisphere (21), sediments (21), and seawater (7) were extracted using the DNeasy PowerSoil Kit (Qiagen, Germany). PCR amplification of bacterial and archaeal 16S rRNA gene fragments was performed with primers 515F (5’-GTGCCAGCMGCCGCGGTAA-3′) and 806R (5’-GTGCCAGCMGCCGCGGTAA-3′) [34] and with primers 787F (5’-ATTAGATACCCSBGTAGTCC-3′) and 1059R (5’-GCCATGCACCWCCTCT-3′) [35], respectively. Amplicons were purified and checked for quality using a NanoDrop 2000 spectrophotometer (Thermo Fisher Scientific, USA). The purified PCR products were then used to construct sequencing libraries with the NEBNext Ultra II DNA Library Prep Kit (New England Biolabs, USA), and sequenced on the NovaSeq 6000 platform (Illumina, USA) with 150 bp paired-end reads at Guangdong Magigene Biotechnology Co., Ltd. (Guangzhou, China). Processing of the 16S rRNA gene sequences was performed using Fastp (v0.14.1) [36] and Usearch (v10.0.240) [37]. Quality-filtered paired-end reads were clustered into operational taxonomic units (OTUs) with 97% identity. The clustering process entailed the removal of singleton OTUs and chimera sequences through Usearch. Uparse was employed to select a representative sequence for each OTU. Taxonomic information was assigned to individual OTUs by aligning them to the SILVA 138 reference database, with those OTUs annotated as chloroplasts and mitochondria being excluded.

### Metagenomic sequencing

DNA from 21 plastisphere samples was used for shotgun metagenome library construction. The sequencing libraries were generated using an NEBNext Ultra II DNA Library Prep Kit (New England Biolabs, USA), following the manufacturer’s instructions. The libraries were sequenced on the NovaSeq 6000 platform (Illumina) at Guangdong Magigene Biotechnology Co., Ltd. (Guangzhou, China) using 150 bp paired-end reads. The raw reads were processed using Trimmomatic (v.0.36) to obtain clean data [38]. The filtered reads were then assembled using MEGAHIT (v1.2.9) with the parameters “k-min 35, k-max 115, k-step 20” [39]. Contigs ≥500 bp were used for the prediction of open reading frames (ORFs) with MetaGeneMark (v3.38) using default parameters. Redundancy was removed using CD-HIT (v4.7) to obtain a unique initial gene catalog by clustering at 95% identity with 90% coverage and using the longest contig as the representative sequences. Read mapping of the non-redundant sequences was conducted with BBMap (v38.90) using the default parameters. The non-redundant sequences were searched against the NCBI-nr database to obtain their taxonomic assignment. The abundance of *amoA* genes in each sample was calculated as the number of mapped reads divided by the length of *amoA* genes.

### Metagenomic binning and analysis

Contigs with a length of at least 2000 bp were retained and binning was performed using MetaWRAP v.1.2.1 by three binning methods (CONCOCT v.0.4.0, MaxBin v.2.2.2, and MetaBAT v.2.12.1) [40]. CheckM v1.0.12 was utilized to evaluate the completeness and contamination of the refined metagenome-assembled genomes (MAGs) [41]. Further analysis was carried out only on MAGs that met the criteria of medium to high quality (i.e., ≥ 50% complete and < 10% contamination). The taxonomic classification of the acquired MAGs was conducted using GTDB-tk v1.7.0 [42]. Prodigal utilized the “-p meta” parameters to translate the MAGs and the predicted ORFs were annotated using the KEGG Automatic Annotation Server (www.genome.jp/tools/kaas/).

The number of dereplicated MAGs was determined using Bowtie2 (v2.2.8) by aligning the high-quality reads to the contigs of the MAGs [43]. Coverage values for the contigs were calculated using BEDTools. The relative abundances (percentage) of the obtained MAGs were determined by dividing the total coverage of contigs belonging to each MAG by the total coverage of all contigs, then multiplying by 100. The average amino acid identity (AAI) between MAGs and chosen reference genomes was computed using CompareM (v0.1.2).

### Phylogenetic tree construction

For phylogenetic analysis of AOA, AOB, and CMX *amoA* genes, the metagenomic unigene sequences identified as EC.1.14.99.39 by KEGG were extracted. Nucleotide sequences of the 12 *amoA* genes recovered in this study and those of 28 reference *amoA* genes (Table S1) were aligned using MAFFT (v7.407) [44], and further edited by trimAl [45]. A maximum-likelihood phylogenetic tree was constructed using IQ-TREE (v1.6.10) [46] with the option “-bb 1000”. The tree was further annotated using the iTOL tool [47]. Two MAGs from each group (AOA, AOB, and CMX) were obtained in this study, and phylogenetic analysis of the six MAGs was performed using PhyloPhlAn (v3.0.3) [48]. The genome sequences of 16 AOA, 10 AOB, or 21 CMX were used as references (Table S2). The resulting ML trees were visualized using iTOL tool [47].

### *amoA* gene abundance

AOA, AOB, and CMX *amoA* genes were used as functional and phylogenetic markers to identify and quantify ammonia oxidizers. Archaeal, bacterial, and comammox *amoA* genes were quantified using qPCR with respective domain-specific primers of Arch-*amoA*-F/Arch-*amoA*-R [49], *amoA*-F/*amoA*-R [50], and Ntsp-*amoA*-162F/Ntsp-*amoA*-162R [51]. The standard curves were obtained using a 10-fold serial dilution of a plasmid containing the AOA/AOB/CMX target gene inserts and had amplification efficiencies ranging from 93% to 96%, correlation coefficients (*R*^2^) above 0.98, and values exceeding 1.0 × 10^2^ copies g^−1^. Detailed information of the primers is given in Table S3.

### Statistical analysis

Statistical analyses were performed using the SPSS Statistical Package version 16.0 (SPSS, Inc., Chicago, USA). One-way ANOVA was used to detect the significant differences between samples at different time points during colonization and between plastisphere and sediments for various parameters. Fisher’s least significant difference test was also employed to determine significant differences among AOA, AOB, and CMX for parameters measured in the nitrification experiment. Pearson correlation analysis was used to determine significant correlations between the relative abundances of individual ammonia-oxidizers and various environmental factors. A *P* value <0.05 was considered statistically significant.

## Results

### Temporal dynamics of EPS in the plastisphere

**Figure 1:**
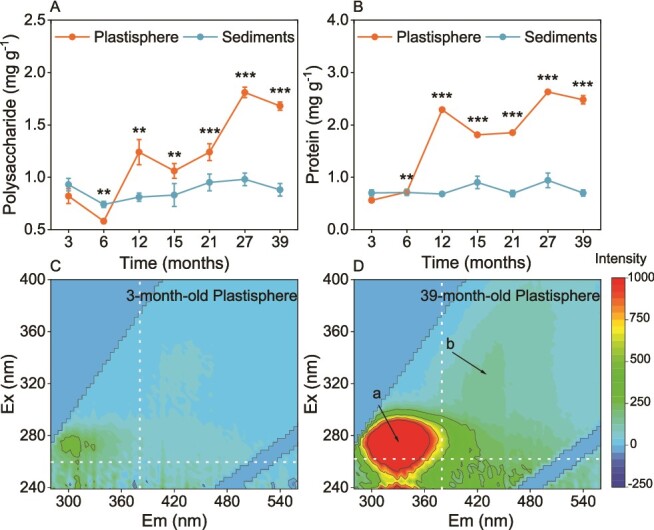
**Temporal dynamics of extracellular polymeric substances (EPS) in the plastisphere.** (a and B) variations in the concentration of polysaccharide (PS) and protein (PN) of EPS during the experimental period, respectively. Error bars represent SD (*n* = 3). For the plastisphere, significant differences between the 3-month-old samples and those from other time points are indicated by asterisks: ^*^P < 0.05; ^*^^*^P < 0.01; ^*^^*^^*^P < 0.001; for sediments, relatively little variation was observed and is thus not indicated. (C and D) three-dimensional excitation-emission matrix fluorescence spectra of EPS in the 3- and 39-month-old plastisphere, respectively. Peak a and b in panel D were identified as aromatic protein-like and humic acid-like substances, respectively.

Concentrations of PS and PN in the plastisphere showed an increasing trend over the 39-month experimental period with 1.68 and 2.48 mg g^−1^ in the 39-month-old plastisphere, and which were two and four times higher than the 3-month-old plastisphere, respectively (Fig. 1A and 1B). EEM analysis revealed that the intensities of fluorescent peaks a (275/340–350 nm) and b (250–320/440 nm) of EPS in the 39-month-old plastisphere were higher than those in the plastisphere F 27-month-old and the 39-month-old sediments (Figs. 1C, 1D, and S5). Peaks a and b were identified as aromatic protein-like substances and humic acid-like substances, respectively [52]. In contrast, concentrations of both PS and PN in sediments remained consistently low over the entire colonization experiment except for a decrease in PS at month 6 (Fig. 1A and 1B).

### Temporal dynamics of the microbial community of the plastisphere

**Figure 2:**
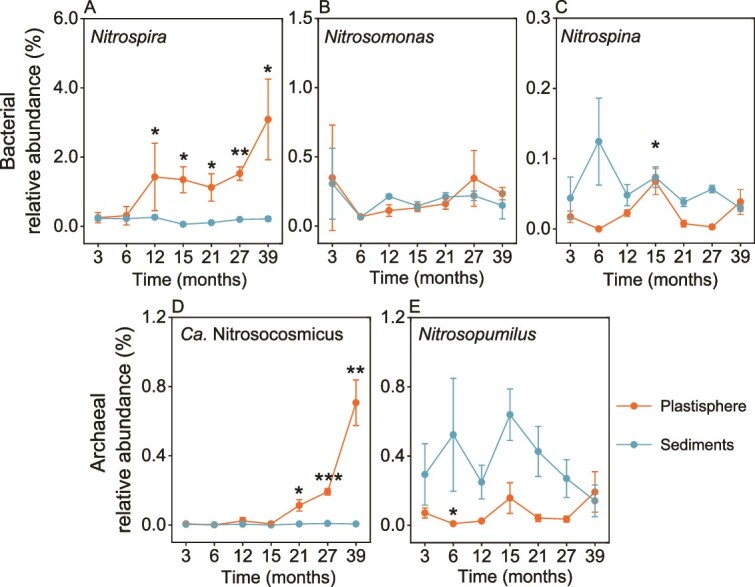
**Temporal dynamics of the relative abundances of nitrifiers in the plastisphere based on 16S rRNA gene sequencing.** (A-C) variations in the relative abundances of *Nitrospira*, *Nitrosomonas,* and *Nitrospina* in the plastisphere and sediments, respectively. (D-E) variations in the relative abundances of Ca. Nitrosocosmicus and *Nitrosopumilus* in the plastisphere and sediments, respectively. For the plastisphere, significant differences between the 3-month-old samples and those from other time points are indicated by asterisks: ^*^P < 0.05; ^*^^*^P < 0.01; ^*^^*^^*^P < 0.001; for sediments, relatively little variation was observed and is thus not indicated.

16S rRNA gene amplicon sequencing revealed that at the phylum level, nitrifying *Nitrospirota* bacteria and *Nitrososphaerota* archaea accumulated gradually in the plastisphere over the 39-month colonization period (Figs. S6 and S7). At the genus level, the relative abundance of *Nitrospira*, a member of the *Nitrospirota* phylum, increased steadily from 0.3% at month to 3.2% at the end of the experiment (39 months), indicating a 10-fold increase (Fig. 2A). The relative abundance of Ca. Nitrosocosmicus, a member of the *Nitrososphaerota* phylum, increased significantly (*P* < 0.05) from 0.02% at month 15 to the final value of 0.7%, showing a 35-fold increase (Fig. 2D). By month 39, Ca. Nitrosocosmicus constituted 80.3% of all archaea (Fig. S8A). In contrast to the plastisphere, the relative abundances of *Nitrospira* and Ca. Nitrosocosmicus in both sediments and seawater remained at low levels (0.2% and 0.004%; 0.1% and 0.004%, respectively) throughout the experiment (Figs. 2A, 2D, and S9). Other nitrifying bacteria (*Nitrosomonas* and *Nitrospina*) and archaea (*Nitrosopumilus*) in the plastisphere, sediments, and seawater exhibited low relative abundances and fluctuated during the experiment (Figs. 2B, 2C, 2E, and S9–S11).

### Metagenomic analysis of ammonia oxidizers in the plastisphere

**Figure 3:**
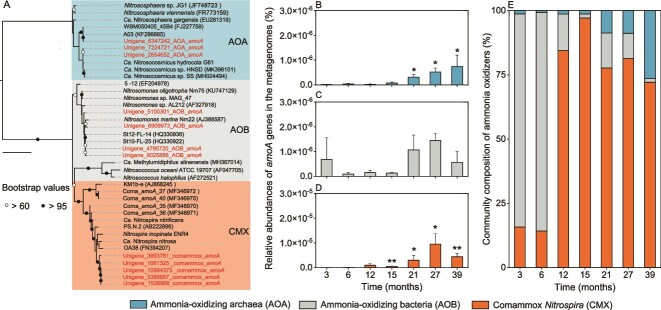
**Temporal dynamics of *amoA* genes of ammonia oxidizers in the plastisphere.** (A) Phylogenetic inference of the 12 *amoA* gene sequences retrieved from the plastisphere metagenomes. The *amoA* genes identified in this study are in red, while reference *amoA* genes are in black. The phylogenetic tree was constructed from 40 *amoA* sequences (860 aligned positions) using maximum likelihood estimation with IQTREE under the TIMe + G4 model. Branch support values were calculated from 1000 replicates and the tree is midpoint-rooted. The scale bar indicates an estimated one nucleotide substitution per site. (B-D) relative abundances of *amoA* genes at different sampling times affiliated with AOA, AOB, and CMX determined by mapping reads to *amoA* sequences in Fig. 3A. Significant differences between the 3-month samples and those from other time points are indicated by asterisks: ^*^P < 0.05; ^*^^*^P < 0.01. (E) Relative abundance AOA, AOB, and CMX *amoA* genes in metagenomes at different timepoints.

Among the 12 *amoA* genes identified, 3, 4, and 5 were affiliated with AOB (*Nitrosomonas*), AOA (*Nitrososphaeraceae*), and CMX, respectively (Fig. 3A). The relative abundances of *amoA* genes affiliated with AOA and CMX exhibited an increasing trend with time (Fig. 3B and 3D). The relative abundance of AOA-affiliated *amoA* genes in the 39-month-old plastisphere increased by an order of magnitude compared to that of the 3-month-old plastisphere, while the respective increase of CMX-affiliated *amoA* genes was three orders of magnitude. In contrast, there was no significant difference (*P* > 0.05) in AOB-affiliated *amoA* genes between the 3- and 39-month-old plastisphere (Fig. 3C). In the young plastisphere (≤ 6-months), 51.0% to 78.6% of the retrieved *amoA* genes were affiliated with AOB, while 60.4% to 82.8% were CMX in older samples (≥ 12-months), indicating CMX dominated ammonia oxidizer communities in the old plastisphere. The percentage of AOA-affiliated *amoA* genes to the total *amoA* genes in the plastisphere ranged from 1.8% to 26.0% (Fig. 3E).

**Figure 4:**
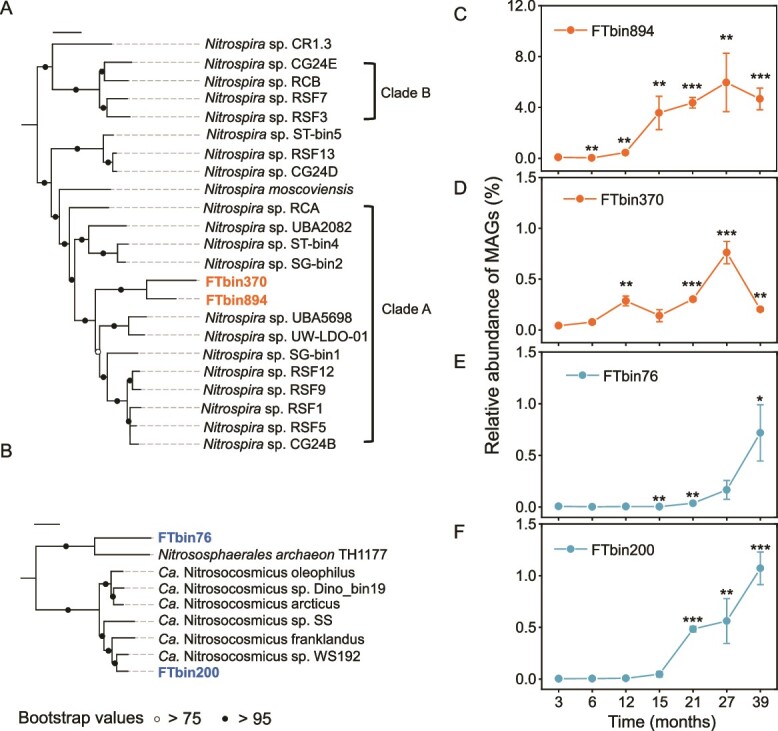
**Phylogenetic affiliation and the relative abundances of four ammonia-oxidizing metagenome-assembled genomes (MAGs) recovered from the plastisphere.** (A) the maximum-likelihood phylogenetic tree was constructed using two CMX MAGs (shown in bold orange) and reference genomes of CMX. Two *Leptospirillum* species from *Nitrospirota* were used as outgroups. The tree was calculated using the LG model from an alignment of 31 483 amino acid positions derived from 349 genes. The tree was constructed by PhyloPhlAn and visualized with iTOL. The scale bar represents an estimated 0.1 average amino acid substitutions per site. (B) Phylogenetic relationships between the two identified AOA MAGs (shown in bold blue) and reference genomes. Two *Nitrosopumilus* species from *Nitrososphaerota* were used as an outgroup. The tree was calculated using the LG model from an alignment of 8217 amino acid positions derived from 140 genes. The scale bars represents an estimated 0.1 amino acid substitutions per site. (C-F) temporal dynamics of the relative abundances of the four abovementioned MAGs in the plastisphere metagenomes, respectively. Significant differences between the 3-month-old samples and those from other time points are indicated by asterisks: ^*^P < 0.05; ^*^^*^P < 0.01; ^*^^*^^*^P < 0.001.

Two MAGs were obtained for each of the three groups, AOA, AOB, and CMX (Figs. 4A, 4B, and S12). The genome sizes of these six MAGs varied from 2.7 to 4.1 Mb, and their GC contents ranged from 29% to 55% (Table S4). A phylogenetic analysis of the two CMX MAGs (i.e., FTbin894 and FTbin370) revealed that they belonged to CMX clade A. The relative abundance of FTbin894 increased from 0.08% in the 3-month-old plastisphere to 4.7% in the 39-month-old plastisphere, while the increase of FTbin370 at the same period varied from 0.04% to 0.8% (Fig. 4C and 4D).

One AOA MAG (FTbin76) had a close phylogenetic relationship with *Nitrososphaerales archaeon* TH1177 (Fig. 4B). However, the pairwise average amino acid identities (AAI) between the other AOA MAG (FTbin200) and the 23 publicly available genomes of Ca. Nitrosocosmicus were ≤ 88.8% (Table S5), indicating that the MAG can be considered a new species [53]. Similar to CMX MAGs, the relative abundances of the two AOA MAGs increased constantly over the 39-month experimental period (Fig. 4E and 4F). The relative abundances of FTbin76 and FTbin200 increased from 0.007% and 0.003% in the 3-month-old plastisphere to 0.7% and 1.1% in the 39-month-old plastisphere, respectively. Unlike CMX and AOA MAGs, the relative abundances of the two AOB MAGs (FTbin20 and FTbin153) did not increase with time (Fig. S12).

### Relationships between environmental factors and ammonia oxidizers

**Figure 5:**
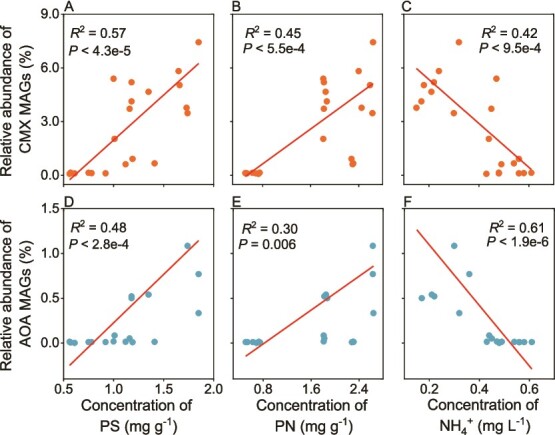
**Pearson correlations between the relative abundances of ammonia oxidizers and the concentrations of EPS components in the plastisphere and NH**_**4**_^**+**^ **in seawater.** (A-C) Pearson correlations between the relative abundance of the two CMX MAGs and the concentration of polysaccharides (PS), proteins (PN), and NH_4_^+^, respectively. (D-E) Pearson correlations between the relative abundance of the two AOA MAGs and the concentration of PS, PN, and NH_4_^+^, respectively. Pearson’s correlation coefficients and *P* values are given in the panels.

The relative abundance of the CMX MAGs and AOA MAGs positively correlated with PS and PN concentrations but negatively with NH_4_^+^ concentration in the plastisphere (*P* < 0.05; Fig. 5). In contrast, there were no significant correlations between the relative abundance of the AOB MAGs and these three parameters (data not shown). When the relative of the six ammonia oxidizer MAGs were analyzed individually, similar trends were obtained (Fig. S13A). In agreement with the above-mentioned results, the relative abundances of *Nitrospira* and Ca. Nitrosocosmicus were positively correlated with PS and PN concentrations but negatively correlated with NH_4_^+^ concentration (*P* < 0.05; Fig. S13B).

### Nitrification activity and N_2_O production of the 39-month-old plastisphere

**Figure 6:**
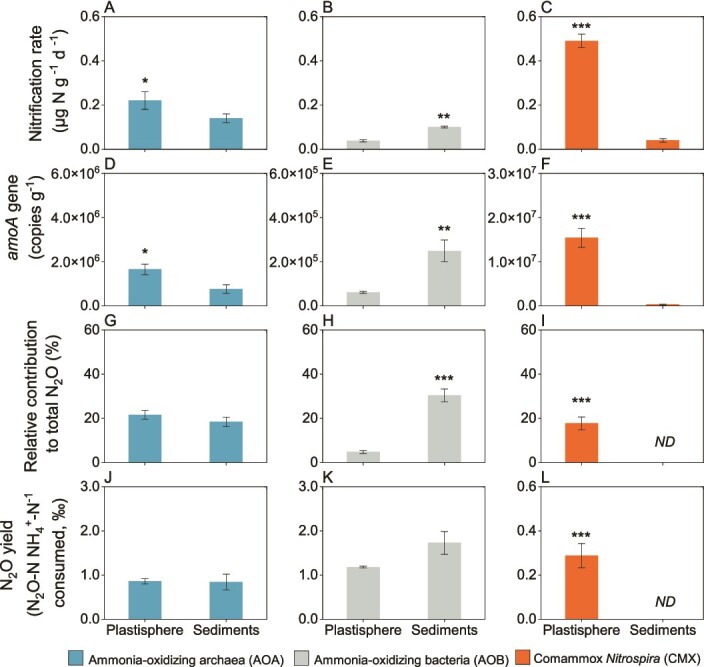
**Contributions of ammonia oxidizer groups to nitrification and N**_**2**_ **O production in the 39-month-old plastisphere.** (A-C) nitrification rates attributed to AOA, AOB, and CMX. (D-F) abundance of *amoA* genes determined for qPCR analysis specific for AOA, AOB, and CMX. (G-I) contributions of the three major types of ammonia oxidizers to total N_2_O emission. (J-L) N_2_O yields of AOA, AOB, and CMX. Significant differences between the plastisphere and sediments for the same type of ammonia oxidizer are indicated by asterisks: ^*^P < 0.05; ^*^^*^P < 0.01; ^*^^*^^*^P < 0.001.

In the 39-month-old plastisphere, the nitrification rate of CMX was 0.49 μg N g^−1^ d^−1^ (Fig. 6C), which was significantly higher than that of AOA (0.22 μg N g^−1^ d^−1^; Fig. 6A) and AOB (0.04 μg N g^−1^ d^−1^; Fig. 6B). Consistent with this pattern, the abundance of CMX *amoA* genes in the 39-month-old plastisphere microcosm (1.5 × 10^7^ copies g^−1^; Fig. 6F) were 9- and 250-fold greater than those of AOA (1.6 × 10^6^ copies g^−1^; Fig. 6D) and AOB (6.1 × 10^4^ copies g^−1^; Fig. 6E). In the sediments, the nitrification rates and *amoA* gene abundances of CMX and AOA were significantly lower than those in the 39-month-old plastisphere, but an opposite trend was observed in the nitrification rate and *amoA* gene abundance of AOB (Fig. 6A–6F).

The total N_2_O emissions of the microcosms containing 39-month-old plastisphere and sediments over the 3-day experimental period were 2.52 and 1.81 ng N_2_O N g^−1^, respectively. After the addition of acetylene for inhibiting ammonia oxidation, N_2_O emissions derived from non-ammonia oxidation processes in these two microcosms were 1.41 and 0.93 ng N_2_O N g^−1^, comprising 55.9% and 51.7% of the total N_2_O emissions, respectively. In other words, 44.1% and 48.3% of the total N_2_O emissions in the plastisphere and sediment microcosms were attributed to nitrification, respectively. Specifically, in the sediment microcosms, AOA and AOB contributed 18.3% and 30.0% of the total N_2_O emission, with CMX having no contribution (Fig. 6G–6I). In contrast, 21.4%, 17.9%, and 4.8% of the total N_2_O emissions in the plastisphere microcosm were related to AOA, CMX, and AOB, respectively (Fig. 6G–6I). The N_2_O yields for AOA, AOB, and CMX were calculated as their respective N_2_O-N (ng) emission divided by NH_4_^+^-N oxidized. The N_2_O yields for AOA and AOB in the plastisphere microcosm were 0.8‰ and 1.2‰, respectively, and both were significantly higher than that for CMX (0.3‰) (Fig. 6J–6L, and Table S6). The cell-specific N_2_O production rate (i.e., the activity per cell) was inferred from the calculated N_2_O emission rate and *amoA* gene abundance, which can be used as a proxy for cell numbers [54]. In the plastisphere, the cell-specific N_2_O production rate of CMX (0.03 amol cell^−1^ h^−1^) was much lower than those of AOA (0.3 amol cell^−1^ h^−1^) and AOB (4.6 amol cell^−1^ h^−1^; Table S7).

## Discussion

### Temporal enrichment of CMX and Ca. Nitrosocosmicus in the plastisphere

Members of CMX have been detected in various ecosystems, including hot springs [55], rivers [56], aquaculture pond waters [57], and bioreactors [58–60]. However, it still remains unclear whether CMX can colonize the surface of coastal plastic debris. The colonization of the coastal plastisphere by CMX was revealed by the recovery of both *amoA* genes and MAGs, with their relative abundance increased over time and CMX dominating the ammonia-oxidizing community after colonization for 12 months or more (Figs. 3 and 4). These results indicate that CMX were not only enriched but also outnumbered AOA and AOB over a long-term succession of the plastisphere (> 1 year). Similarly, two recent studies lasting about 16 months also reported the temporal enrichment of CMX and their dominance in the ammonia-oxidizing community in a urine-fed membrane bioreactor [58] and a moving bed biofilm reactor [59]. The previous findings, together with our results, highlight the importance of examining the long-term dynamics of different kinds of ammonia oxidizers in microbial-mediated nitrification studies in various ecosystems.

In marine environments, AOA are often more abundant than AOB [61, 62] and our qPCR analyses similarly revealed that AOA-*amoA* genes in the 39-month-old plastisphere were approximately 25 times higher than that of AOB (Fig. 6). Read mapping to *amoA* genes and *amoA*-carrying MAGs further indicated that the relative dominance of AOA and AOB varied over a long period (Figs. 3 and 4). A focused analysis of AOA communities in the plastisphere, sediments, and seawater revealed the temporal enrichment of Ca. Nitrosocosmicus in the plastisphere but not in the other two habitats. After 39 months of colonization in the plastisphere, the relative abundance of Ca. Nitrosocosmicus in the archaeal community (80.3%) was much higher than that of *Nitrosopumilus* (1.2%), implying that the plastisphere favored the growth of Ca. Nitrosocosmicus rather than *Nitrosopumilus* (Fig. S8). This contrasts with previous studies identifying Ca. Nitrosocosmicus strains in terrestrial environments, such as soils [63, 64], freshwater sediments [65], and wastewater treatment plants [66].

### Changes in EPS and NH_4_^+^ concentration correlated with temporal dynamics of CMX and Ca. Nitrosocosmicus

The relative abundances of CMX and Ca. Nitrosocosmicus in the plastisphere correlated positively with PS and PN concentrations, indicating the accumulation of EPS in the plastisphere promoted the growth of these ammonia oxidizers. Previous studies reported that EPS constitutes over 90% of the dry weight of most biofilms, contributing to their mechanical stability and enhancing adhesion to surfaces [10, 67]. A higher amount of EPS on the plastic surface can also lead to a thicker biofilm that has a larger oxygen gradient from the surface towards the interior [68]. Our detection of a variety of anaerobic methanogens in the plastisphere suggested that oxygen-limited conditions were present in the biofilm interior (Figs. S8 and S11). CMX encodes bd-like terminal oxidases with a high oxygen affinity, which makes them more competitive in low-oxygen environments [69, 70] and CMX are the dominant ammonia oxidizers in biofilm systems with localized oxygen limitation [71, 72]. Similarly, AOA also have a high affinity for oxygen and are enriched in agricultural soils and marine sediments under low dissolved oxygen conditions [73, 74]. It is likely that the low oxygen level in the interior of biofilm in the plastisphere selects for the temporal enrichment of CMX and Ca. Nitrosocosmicus [75, 76]. In addition, the specific effect of PN (a hydrophobic component of EPS) on the enrichment of Ca. Nitrosocosmicus merits further investigation, given that the cells of Ca. Nitrosocosmicus isolated from an oil-affected environment [63] showed high hydrophobicity and that PN of the EPS in the plastisphere tended to increase with time.

The observed negative correlations between ammonium concentration in the seawater and the relative abundances of CMX and Ca. Nitrosocosmicus in the plastisphere suggested that ammonia in the environment is a major factor influencing the temporal dynamics of CMX and Ca. Nitrosocosmicus. The affinities of different ammonia oxidizers for ammonia have been regarded as a primary determinant of their competition and niche partitioning, as they all utilize ammonia as an energy and nitrogen source for growth [23, 61, 77]. The dominance of CMX in ammonia-oxidizing communities inhabiting environments with low ammonium levels, such as some agricultural soils with 0.07–0.82 mg NH_4_^+^ kg^−1^ [78], groundwater rapid sand filters with 0.53 mg NH_4_^+^ L^−1^ [51], and drinking water treatment plants with 0.05 mg NH_4_^+^ L^−1^ [79] suggested the possible existence of certain CMX having high affinity for ammonia. Our study not only showed the dominance of CMX in most ammonia-oxidizing communities inhabiting the plastisphere but also revealed their dominance increased with overall decreasing ammonium concentration in the surrounding seawater with NH_4_^+^ concentrations ranged from 0.15 to 0.58 mg L^−1^ (overall decline with some fluctuations, Fig. S14A). Some members of Ca. Nitrosocosmicus have been reported to adapt to low ammonia concentrations and outcompete other ammonia oxidizers in ammonia-limited environments [80]. However, the ammonia affinities of the cultured representatives of *Nitrosocosmicus* genus from terrestrial environments were lower than those of other characterized AOA and comparable to previously determined ammonia affinities of AOB [63, 65, 66]. Such a discrepancy may be due to the changes in genetic and physiological properties during the selective isolation of Ca. Nitrosocosmicus strains in the laboratory with an unlimited supply of ammonia [27]. Moreover, the ammonia affinities of laboratory cultures may differ from those in “natural” environments with frequent exposure to fluctuating substrate concentrations [27]. There is no indication that high ammonia tolerance is necessarily linked to low ammonia affinity [76].

### CMX and AOA were major drivers of nitrification in the plastisphere

Nitrification is a pivotal component of the global biogeochemical nitrogen cycle [81]. There was evidence that CMX played a dominant role in the nitrification processes in some natural and engineering environments, such as agricultural soils [32], bioreactors [72], and a riparian ecosystem [82]. In this study, the nitrification rate of CMX in the 39-old-month plastisphere was up to 2–10 times higher than those of AOA and AOB, suggesting that CMX were a major driver of the nitrification process occurring in the plastisphere and was consistent with their dominance of the ammonia-oxidizing community revealed by metagenomic analysis and *amoA* gene abundance. In this context, we could expect that CMX were also a major driver of nitrification in the 12- to 27-month-old plastisphere where they were similarly dominant (Fig. 3), and indicate that CMX-mediated nitrification may be an important component of the biogeochemical cycling of nitrogen in coastal environments.

AOA have been proposed to play a more important role than AOB in ammonia oxidation in the global ocean [61, 83] and coastal environments [26, 84] and nitrification rates of AOA in the 39-old-month plastisphere and sediments were also higher than those of AOB in this study. Nitrification rates in the 39-month plastisphere were approximately two times higher than the sediments and were consistent with 51x and 2x greater abundance of CMX and AOA *amoA* genes, respectively (Fig. 6). Taken together, these results indicate that the plastisphere, emerging as a novel anthropogenic habitat [4], has an important influence on the nitrification process in coastal environments by selectively enriching CMX and AOA.

### CMX and AOA were important contributors to N_2_O emission of the plastisphere

N_2_O is a greenhouse gas with about 300 times greater radiative forcing than CO_2_, exacerbating climate change, and is predicted to become the dominant ozone-depleting substance in the 21st century [85]. It has been estimated that about half of the total N_2_O emission in global estuarine is produced as a side product of ammonia oxidation [86]. In the present study, the N_2_O produced from ammonia oxidation in the 39-old-month plastisphere and sediments comprised 44.1% and 48.3% of the total N_2_O emissions, respectively. The contribution of CMX-mediated ammonia oxidation to the total N_2_O emission of coastal wetland sediments was proposed to be much lower than that of AOB but higher than that of AOA [26]. However, we found little contribution of CMX-mediated ammonia oxidation to the N_2_O production of the sediments. Such discrepancy may be related to the shorter time period of ammonia oxidation in our study than that of the prior study (three vs. 28 days). Nonetheless, in this study, quantitative analysis of N_2_O emissions related to CMX-mediated ammonia oxidation revealed that CMX contributed 17.9% of the total N_2_O emission from the 39-old-month coastal plastisphere, indicating that the contribution of CMX in the coastal plastisphere to N_2_O emission should not be neglected (Fig. 6). Although the CMX N_2_O yield (0.3‰) of our study seemed to be higher than that of an agricultural soil CMX (0.2‰) [32], the cell-specific N_2_O production rate of CMX (0.03 amol cell^−1^ h^−1^) measured here was lower than 0.5–0.8 amol cell^−1^ h^−1^ previously reported [31, 87], suggesting that the proportionally greater contribution of CMX to N_2_O emission from the coastal plastisphere was largely due to their dominance of the ammonia-oxidizing community. Nevertheless, determining the specific contributions of different ammonia oxidizers to nitrification and N_2_O production derived from nitrification microcosm experiments using a combined inhibitor method should be interpreted with caution, given that the effect of 1-octyne on the growth of CMX could vary with concentration of the inhibitor, incubation time, and habitat [31, 88].

Whilst the abundance and nitrification rate of CMX in the 39-month-old plastisphere were higher than those of AOA, their contribution to the total N_2_O emission (17.9%) was lower than that of AOA (21.4%) (Fig. 6). Furthermore, our results showed that CMX had a lower N_2_O yield and cell-specific N_2_O production rate than AOA, which is consistent with previous findings in some soils [31, 32, 89]. A possible explanation for these findings is that the archaeal ammonia oxidation process couples with nitrite oxidation, making ammonia to N_2_O conversion more efficient since nitrite is both the final product and a substrate for N_2_O production [32]. In contrast, the CMX process completes ammonia to nitrate oxidation in a single microorganism, which may decrease the proportion of N transformed to N_2_O [18, 19].

In conclusion, CMX and Ca. Nitrosocosmicus were temporally enriched in a plastisphere consisting of plastic ropes placed in a mangrove intertidal zone for 39 months with EPS of the plastisphere and ammonium concentration potentially affecting the temporal dynamics of both groups. Both CMX and AOA made a considerable contribution to nitrification and N_2_O emissions of the plastisphere. Such information is critical for understanding the nitrification process in coastal environments polluted by plastics and the potential impacts of the plastisphere on the global nitrogen biogeochemical cycle and climate change.

## Supplementary Material

Supporting_information_wrae186

## Data Availability

The National Omics Data Encyclopedia (NODE, https://www.biosino.org/node/) database contains the raw data for 16S rRNA gene amplicon sequencing in the current study, which can be accessed under Project OEP004485. The metagenomic data and MAGs produced in the present investigation are available for viewing in NODE under Projects OEP004488 and OEP004489, respectively.
