# Supplementary material for: Temporal enrichment of comammox Nitrospira and Ca. Nitrosocosmicus in a coastal plastisphere
Source: ISME J. 2024 Oct 7;18(1):wrae186. doi: 10.1093/ismejo/wrae186 (PMC11471898; doi:10.1093/ismejo/wrae186)
Supplement: Supporting_information_wrae186 [file supporting_information_wrae186.docx]

**Supporting information**

**Temporal enrichment of comammox *Nitrospira* and *Ca.* Nitrosocosmicus in a coastal plastisphere**

Qian Yang^1,2,4,‡^, Yin Zhong^1,2,‡,^*, Shi-wei Feng^3^, Ping Wen^3^, Heli Wang^1,2,4^, Junhong Wu^1,2,4^, Sen Yang^1,2,4^, Jie-Liang Liang^3^, Dan Li^5^, Qiong Yang^6^, Nora F.Y. Tam^7,8^, Ping’an Peng^1,2^

^1^State Key Laboratory of Organic Geochemistry and Guangdong-Hong Kong-Maco Joint Laboratory for Environmental Pollution and Control, Guangzhou Institute of Geochemistry, Chinese Academy of Sciences, Guangzhou 510640, China

^2^Guangdong Key Laboratory of Environmental Protection and Resources and Utilization, Guangzhou 510640, China

^3^Institute of Ecological Science, Guangzhou Key Laboratory of Subtropical Biodiversity and Biomonitoring, Guangdong Provincial Key Laboratory of Biotechnology for Plant Development, School of Life Sciences, South China Normal University, Guangzhou 510640, China

^4^University of Chinese Academy of Sciences, Beijing 100049, China

^5^School of Environment and Civil Engineering, Dongguan University of Technology, Dongguan 523808, China

^6^Guangdong Neilingding Futian National Nature Reserve, Shenzhen 518040, China

^7^School of Science and Technology, Hong Kong Metropolitan University, Hong Kong 999077, China

^8^State Key Laboratory of Marine Pollution and Department of Chemistry, City University of Hong Kong, Hong Kong 999077, China

^‡^Qian Yang and Yin Zhong contributed equally to this work.

^*^Corresponding author: Yin Zhong, Guangzhou Institute of Geochemistry, Chinese Academy of Sciences, Guangzhou 510640, China. E-mail: zhongyin@gig.ac.cn

**Text S1.** **Calculations of inhibitor methods.**

In the treatment I, no inhibitor was added. The NH_4_^+^-N consumption and N_2_O generation were calculated via Equation (1):

*∆NH_4_^+^ or ∆N_2_O _(I)_ = ∆NH_4_^+^ or ∆N_2_O _(CMX)_ + ∆NH_4_^+^or ∆N_2_O _(AOB)_+ ∆NH_4_^+^ or ∆N_2_O _(AOA)_+ ∆ NH_4_^+^ or ∆ N_2_O _(others)_* (1)

In the treatment Ⅱ, AOB was inhibited. The NH_4_^+^-N consumption and N_2_O generation of the treatment Ⅱ was calculated via Equations (2):

∆*NH_4_^+^* or ∆*N_2_O _(Ⅱ)_* = ∆*NH_4_^+^*or ∆*N_2_O _(CMX)_* + ∆*NH_4_^+^* or ∆*N_2_O _(AOA)_* + ∆*NH_4_^+^* or ∆*N_2_O* _(others)_ (2)

In the treatment III, AOB and CMX were inhibited. The NH_4_^+^-N consumption and N_2_O generation of the treatment III was calculated via Equation (3):

∆ *NH_4_^+^* or ∆*N_2_O _(III)_*=∆ *NH_4_^+^* or ∆*N_2_O* _(AOA)_+∆*NH_4_^+^* or ∆*N_2_O* _(others)_ (3)

In the treatment IV, all ammonia oxidation processes were inhibited. Thus, the NH_4_^+^-N consumption and N_2_O production of non-ammonia oxidation process were calculated via Equation (4):

*∆NH_4_^+^ or ∆N_2_O _(Ⅳ)_=∆NH_4_^+^ or ∆N_2_O _(others)_* (4)

∆*NH_4_^+^*or ∆*N_2_O (x)* indicates the utilization of NH_4_^+^ or generation of N_2_O in treatment *x* (varying from Ⅰ to Ⅳ).

combing Equations (1) and (2) yields:

$K_{{NH}_{4}^{+}}$or $K_{{N_{2}O}_{(AOB)}}$=$K_{{NH}_{4}^{+}}$or $K_{{N_{2}O}_{(I)}}$-$K_{{NH}_{4}^{+}}$or$K_{{N_{2}O}_{(II)}}$ (5)

$K_{{NH}_{4}^{+}}$or $K_{{N_{2}O}_{(x)}}$ represents the generation rate of NH_4_^+^ or production rate of N_2_O in 3 days in treatment *x*. $K_{{NH}_{4}^{+}}$or $K_{{N_{2}O}_{(AOB)}}$ represents the potential bacterial ammonia oxidation rate or the N_2_O production rate originating from AOB.

combing Equations (3) and (4) yields:

$K_{{NH}_{4}^{+}}$or $K_{{N_{2}O}_{(AOA)}}$=$K_{{NH}_{4}^{+}}$or$K_{{N_{2}O}_{(III)}}$-$K_{{NH}_{4}^{+}}$or $K_{{N_{2}O}_{( = 4 \backslash* ROMAN \mathrm{IV})}}$ (6)

$K_{{NH}_{4}^{+}}$or $K_{{N_{2}O}_{(AOA)}}$ represents the bacterial ammonia oxidation rate or the N_2_O production rate originating from AOA.

combing Equations (2) and (3) yields:

$K_{{NH}_{4}^{+}}$or $K_{{N_{2}O}_{(CMX)}}$=$K_{{NH}_{4}^{+}}$or$K_{{N_{2}O}_{(II)}}$-$K_{{NH}_{4}^{+}}$or $K_{{N_{2}O}_{(III)}}$ (7)

$K_{{NH}_{4}^{+}}$or $K_{{N_{2}O}_{(CMX)}}$ represents the comammox ammonia oxidation rate or the N_2_O production rate originating from CMX.


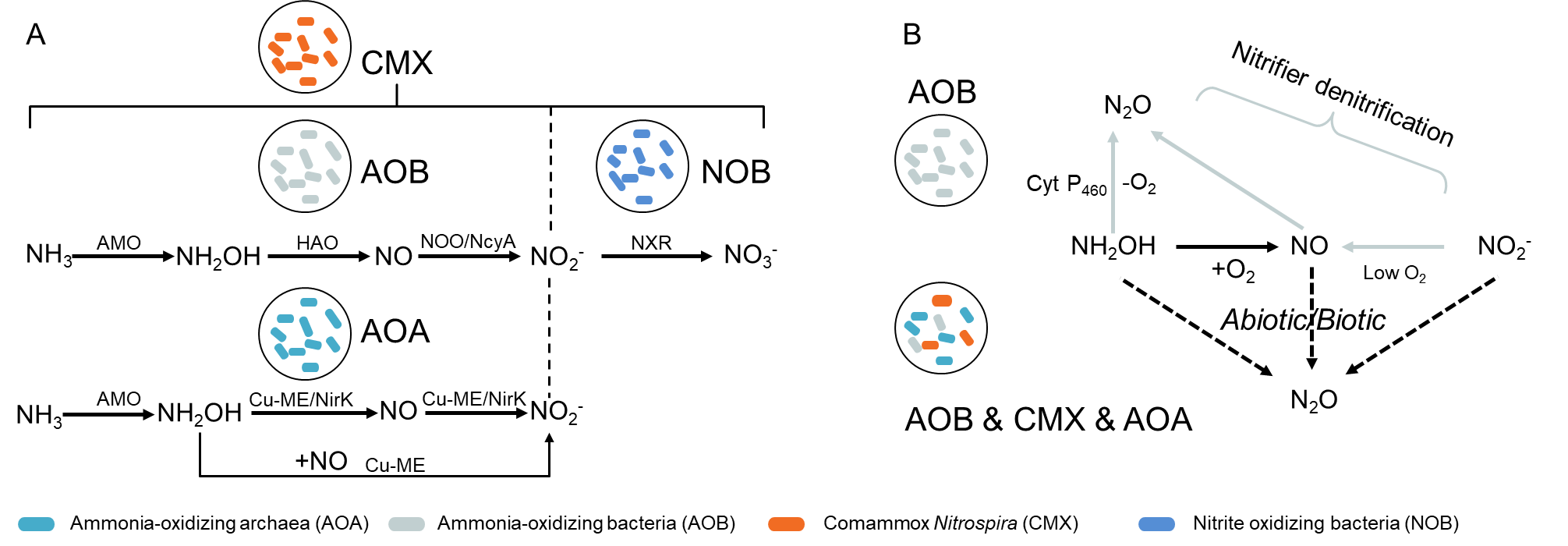


**Fig. S1.** **Nitrification process and N_2_O production during nitrification.** (A) Pathways of nitrification and associated nitrifiers, including CMX: complete ammonia oxidizing bacteria, AOA: ammonia oxidizing archaea, AOB: ammonia oxidizing bacteria, and NOB: nitrite oxidizing bacteria, and associated enzymes, which are AMO: ammonium monooxygenase, HAO: hydroxylamine oxidoreductase, NOO: nitric oxide oxireductase, NcyA: nitrososcyanin, NXR: nitrite oxidoreductase, and NirK: nitrite reductase. Sources: Stein, 2019 [1]. (B) N_2_O production is driven by nitrification processes. Reactions shown in gray arrows are catalyzed only by AOB; reactions with black dashed arrows are abiotic, or biotic via partnering microorganisms, and occur for AOB, CMX, and AOA from intermediates produced during active ammonia oxidation. Sources: Stein, 2019; Han et al., 2021 [1, 2].


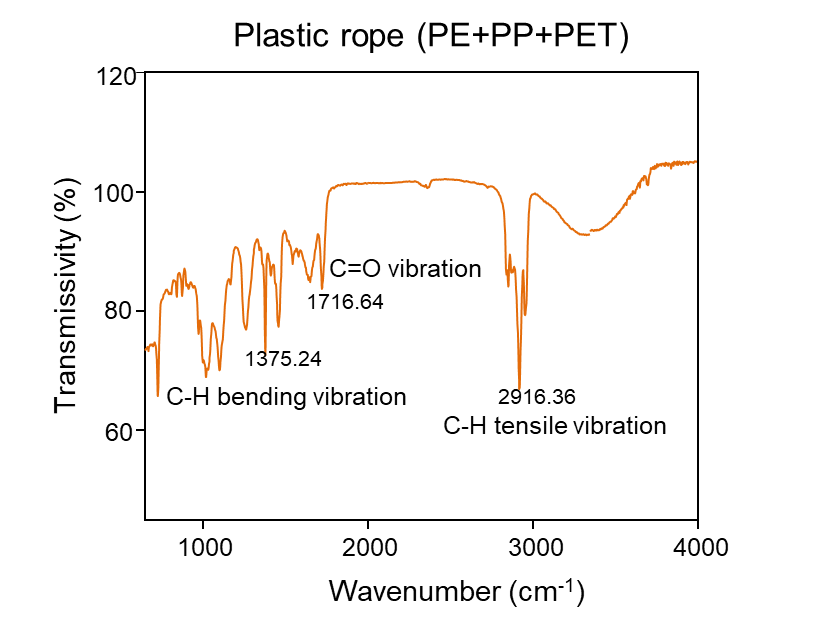


**Fig. S2.** **Fourier-transform infrared spectroscopy (FTIR) analysis of plastic ropes.** Plastic rope is predominantly composed of polyethylene (PE), polypropylene (PP), and polyethylene terephthalate (PET). The main characteristic absorption peak of PET is located at 1716 cm^-1^, which corresponds to the ester carbonyl bond stretching [3]. The PE’s characteristic absorption peaks of 2916 cm^-1^, 720-730 cm^-1^, and 1460-1480 cm^-1^ correspond to the C-H stretching vibration, the bending vibration of CH_2_ groups, and the stretching vibration of CH_2_ groups, respectively [4]. The PP’s characteristic absorption peaks of 2800-3000 cm^-1^, 1375 cm^-1^, and 970-980 cm^-1^ correspond to the C-H stretching vibration, stretching vibration of CH_3_ groups, and a methyl bending vibration, respectively [4].


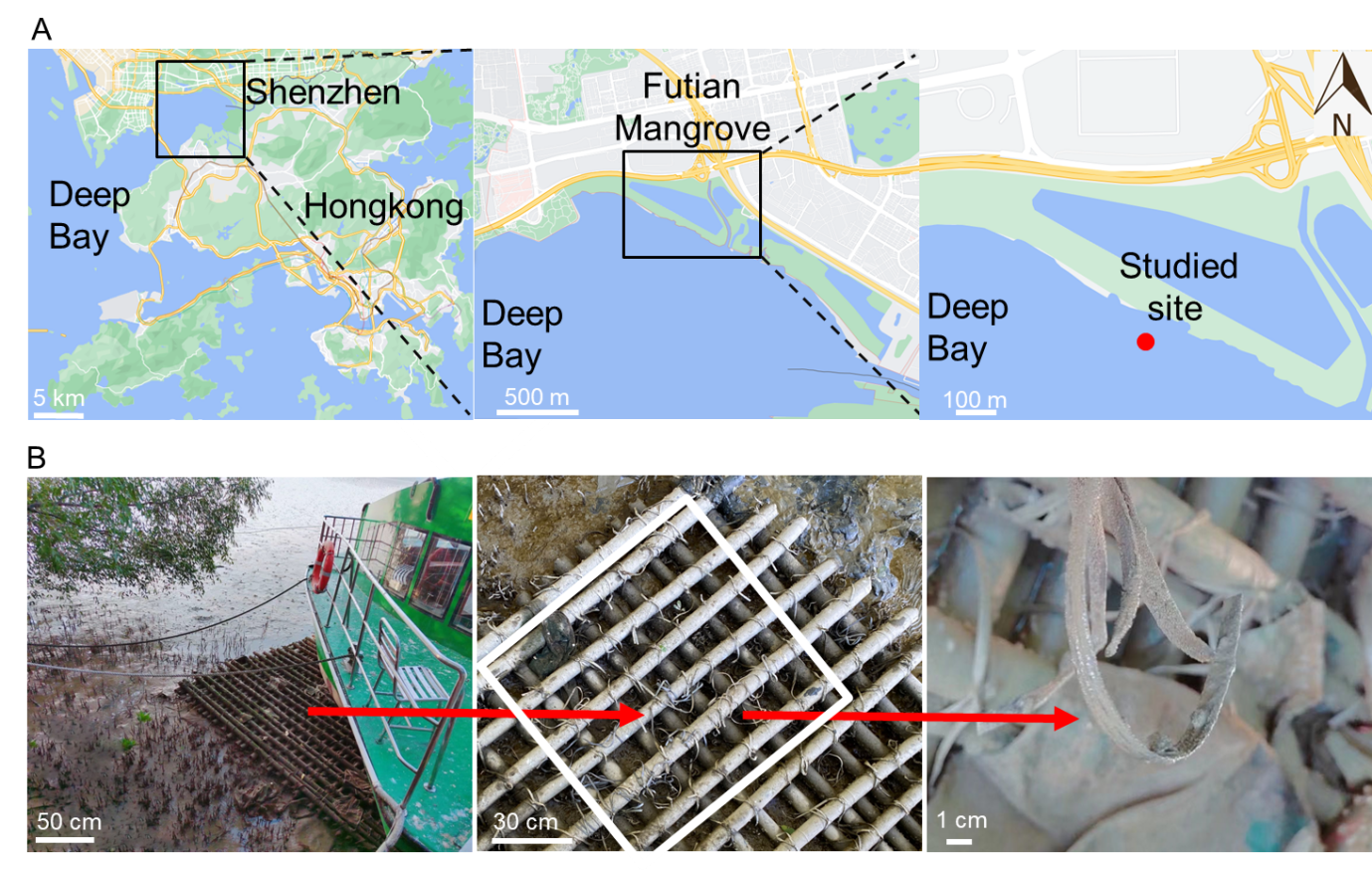


**Fig. S3.** **Location of the study site and schematic diagram of the field colonization experiment.** (A) The studied site is located in the mangrove intertidal zone. (B) Plastic ropes were tied to a bamboo raft.


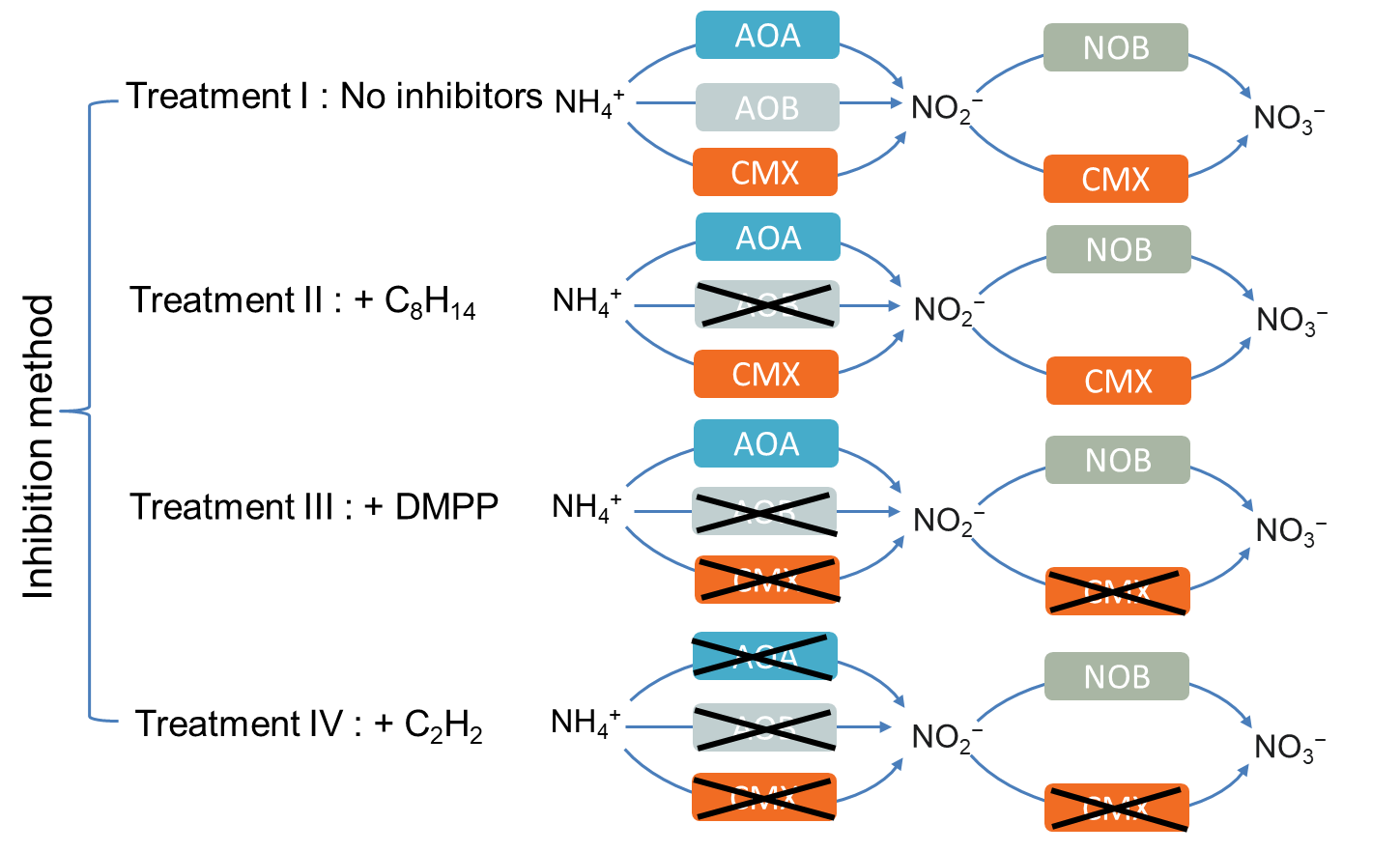


**Fig. S4.** **The schematic diagram of active ingredients in different inhibition treatments.** Treatment I was the control group without any inhibitors; treatment II employed 1-octyne (C_8_H_14_) to inhibit bacterial ammonium oxidation; treatment III utilized 3, 4-Dimethylpyrazole phosphate (DMPP) to inhibit both bacterial ammonium oxidation and comammox, and treatment IV employed acetylene (C_2_H_2_) to inhibit the entire ammonium oxidation process. “AOA,” “AOB”, “CMX” and “NOB” are the abbreviations for ammonia-oxidizing archaea, ammonia-oxidizing bacteria, comammox *Nitrospira*, and nitrite-oxidizing bacteria, respectively.


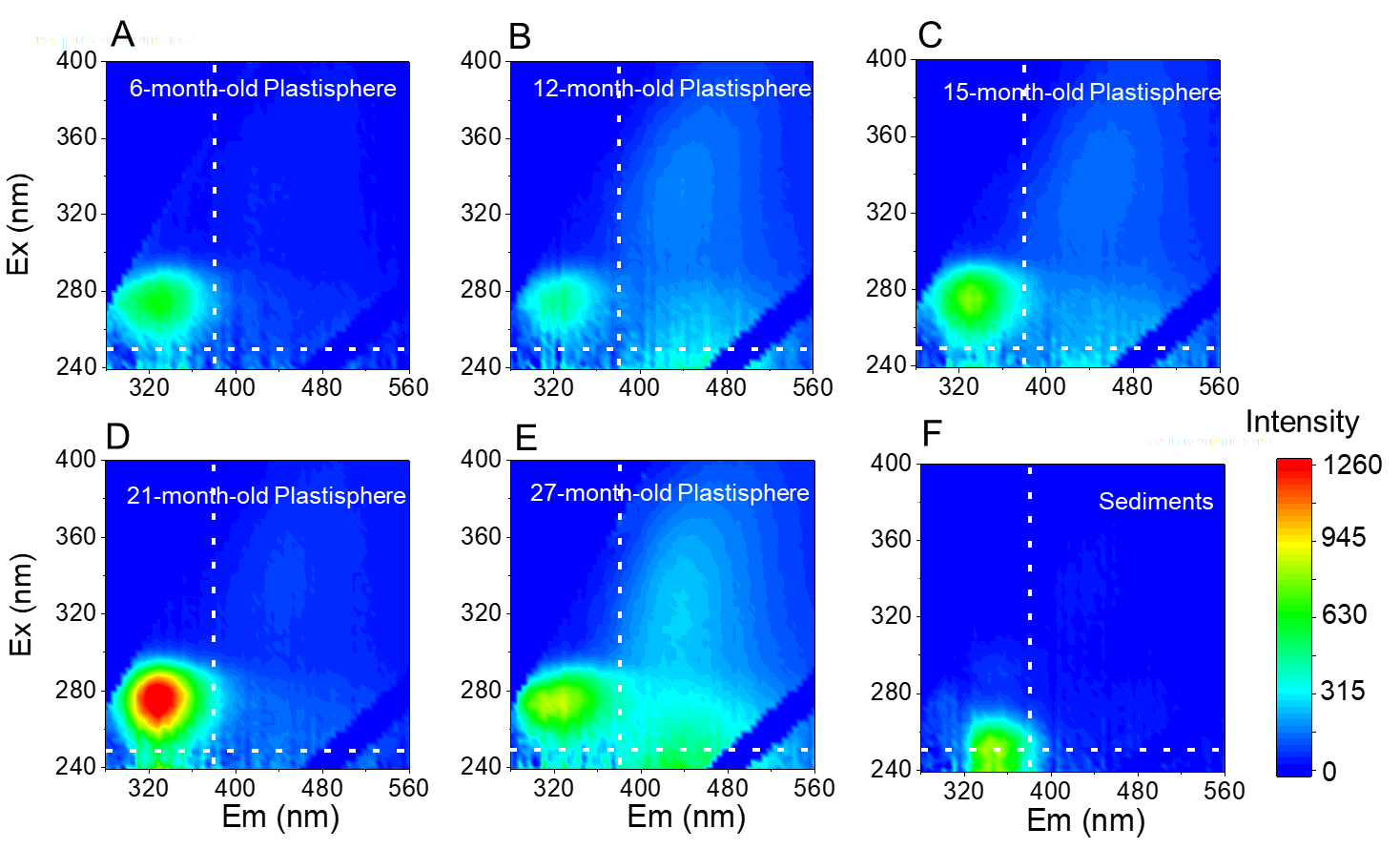


**Fig. S5. EEM fluorescence spectra of EPS of plastisphere and sediments at different colonization time points****. (**A-E) Plastisphere samples at 6, 12, 15, 21, and 27 months, respectively. (F) Sediment sample at 39 months.


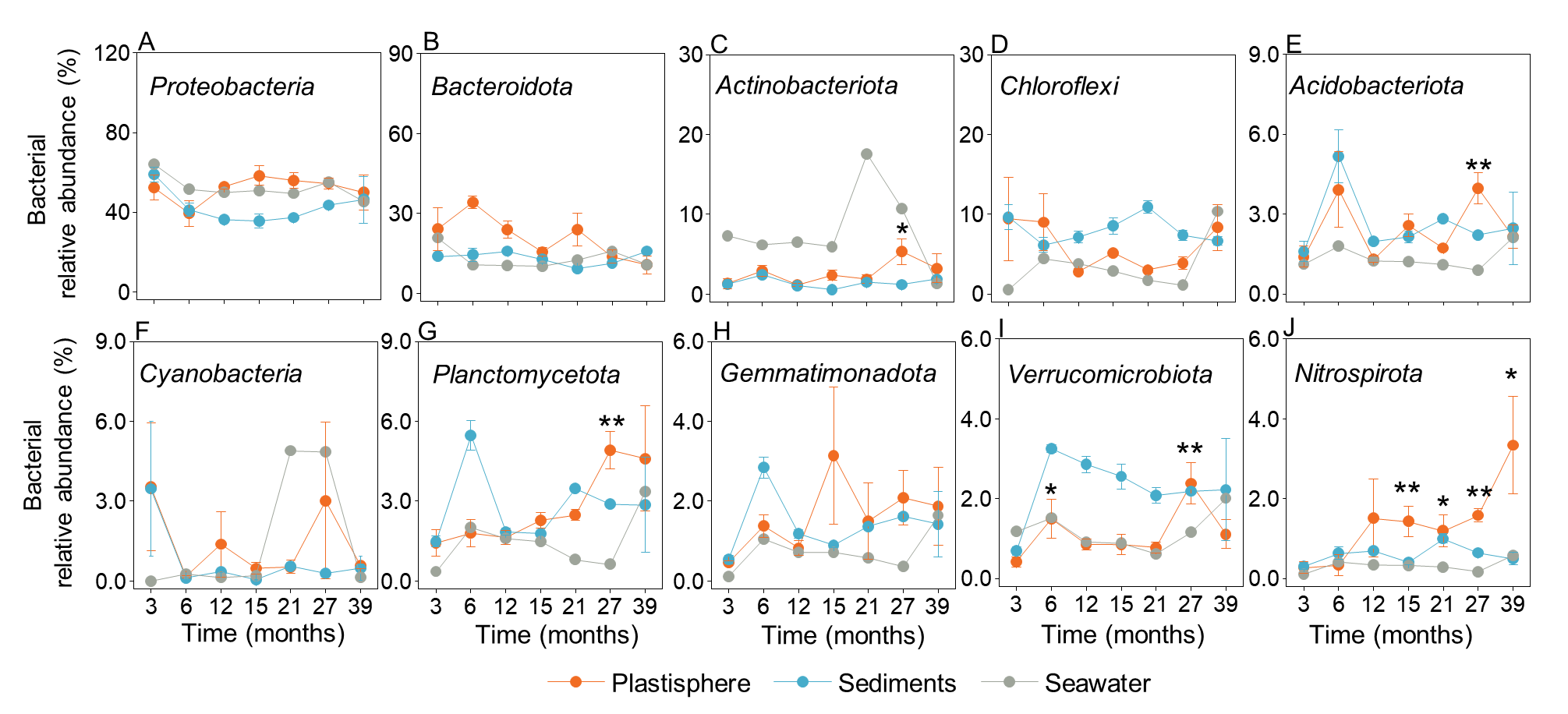


**Fig. S6.** **Variation in the relative abundance of the top 10 bacterial taxa in the plastisphere, sediments, and seawater at the phylum level****.** For the plastisphere, significant differences between the 3-month-old samples and those from other time points were indicated by asterisks: * *P* < 0.05; ** *P* < 0.01; for sediments and seawater, few time variations were found and thus not indicated.


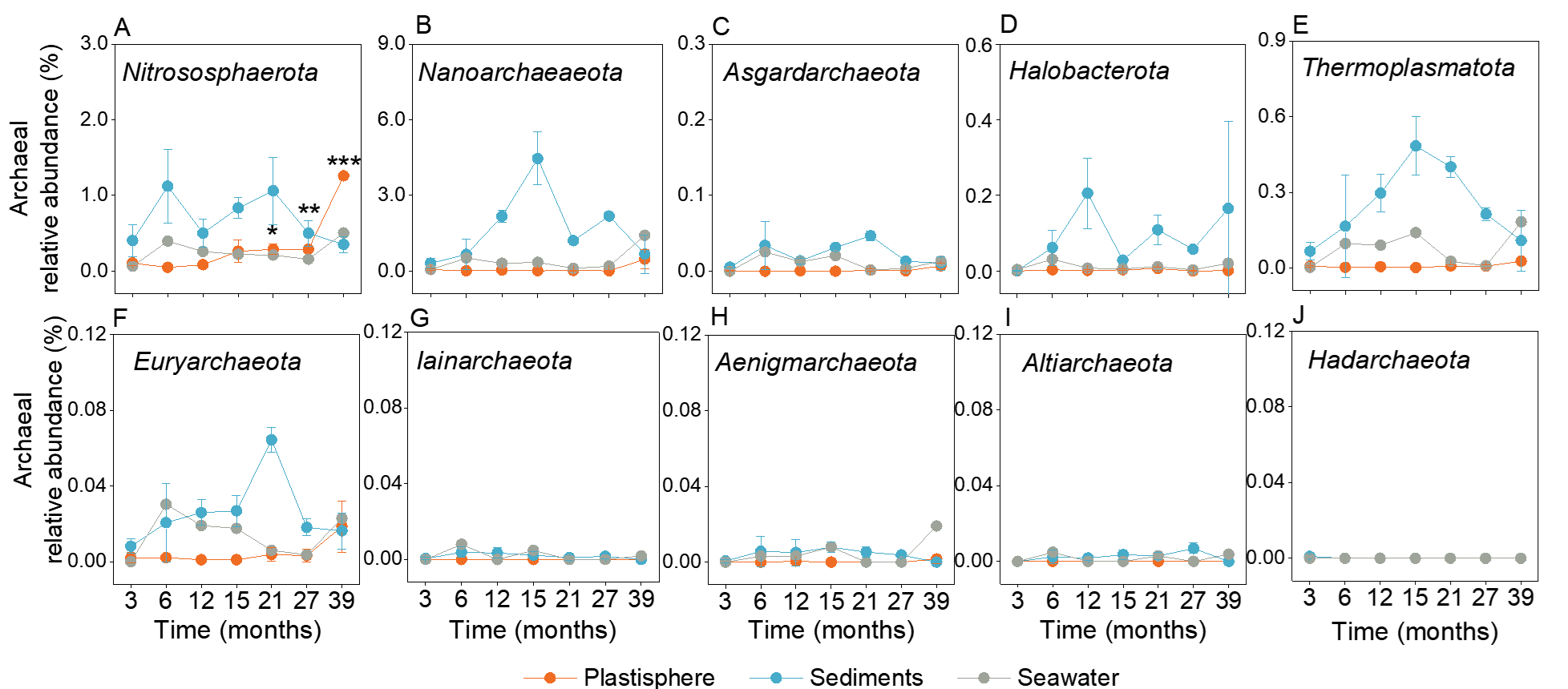


**Fig. S7**. **Variation in the relative abundance of the top 10 archaeal taxa in the plastisphere, sediments, and seawater at the phylum level.** Statistical analysis was performed to assess the significant differences within the plastisphere. Statistical significance is indicated by * *P* < 0.05; ** *P* < 0.01; *** *P* < 0.001, compared with the 3-month-old plastisphere. The archaeal 16S rRNA gene sequences were amplified with primers 515F and 806R.


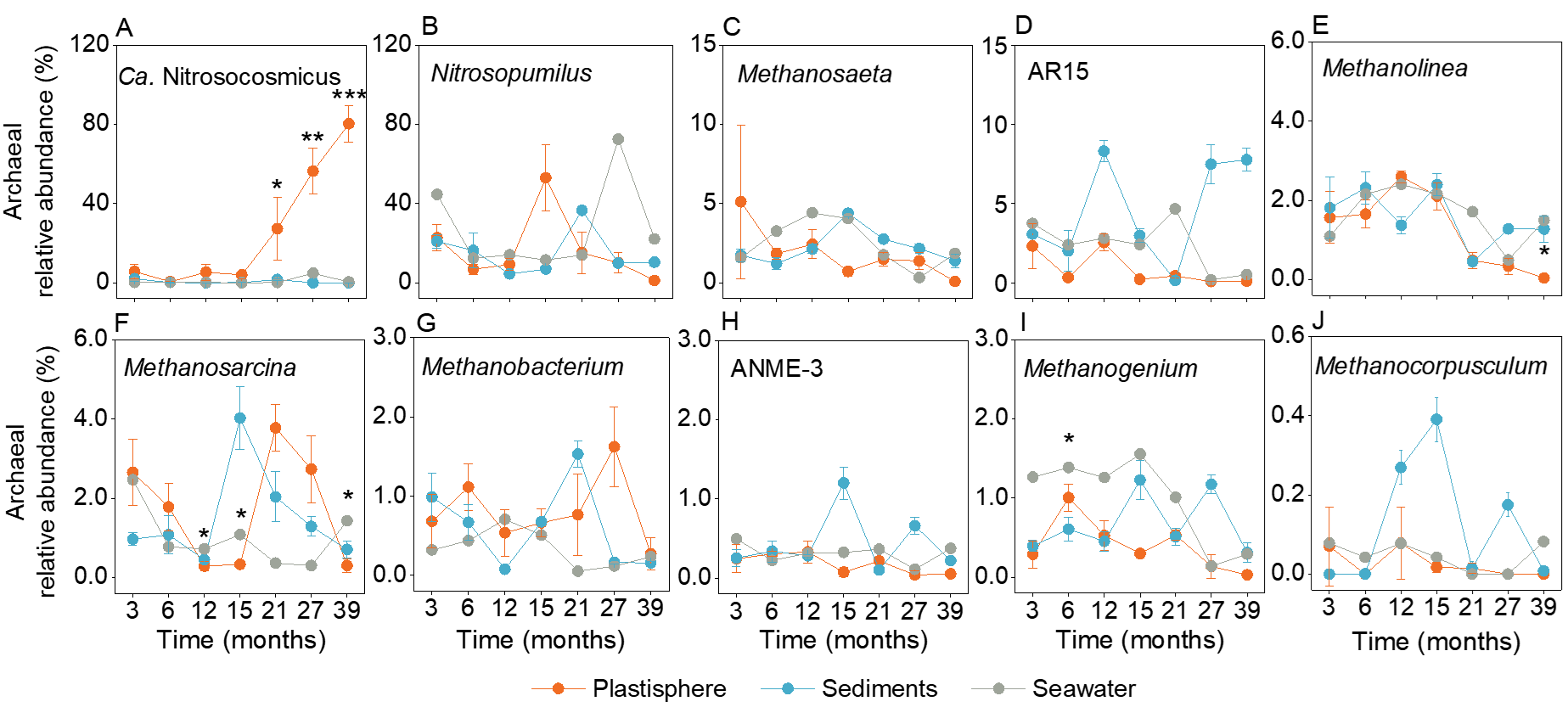


**Fig. S8**. **Time-course variations in** **the relative abundance of the top 10 archaeal taxa in the plastisphere, sediments, and seawater at the genus level.** For the plastisphere, significant differences between the 3-month-old samples and those from other time points were indicated by asterisks: * *P* < 0.05; ** *P* < 0.01; *** *P* < 0.001; for sediments and seawater, few time variations were found and thus not indicated. The archaeal 16S rRNA gene sequences were amplified with primers 787F and 1059R.


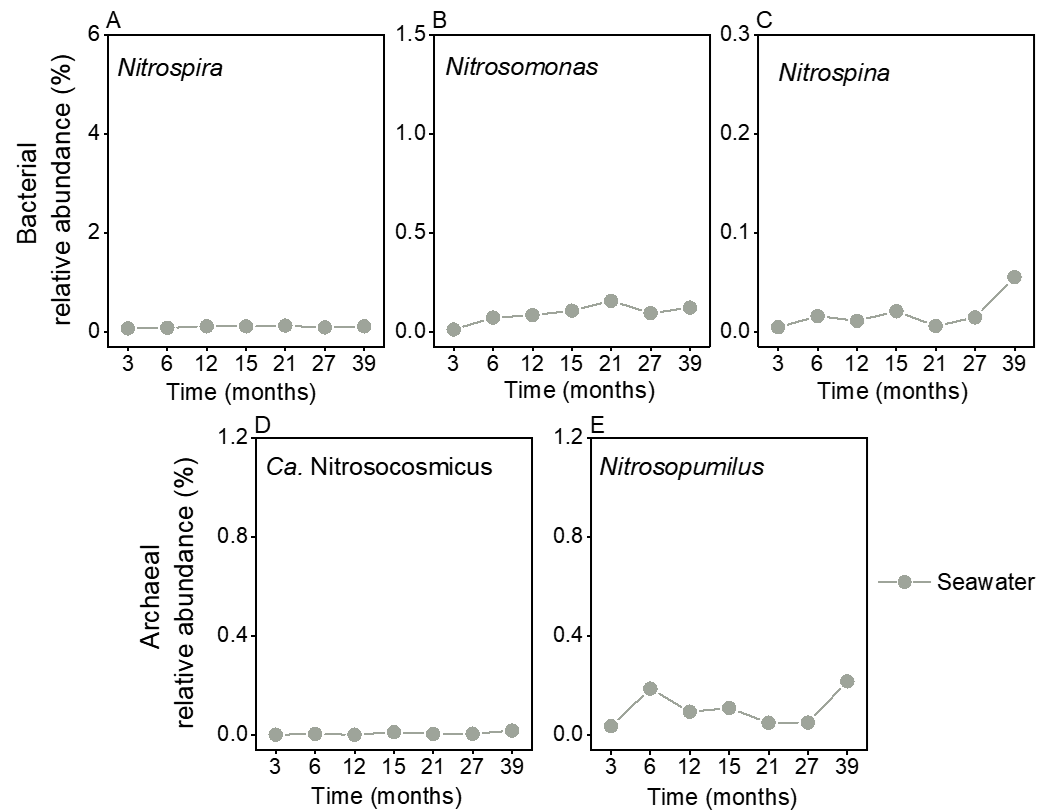


**Fig. S9.** **Variation in the relative abundance of *Nitrospira* (A), *Nitrosomonas* (B), *Nitrospina* (C), *Ca*. Nitrosocosmicus (D), and *Nitrosopumilus* (E)** **in the seawater.**


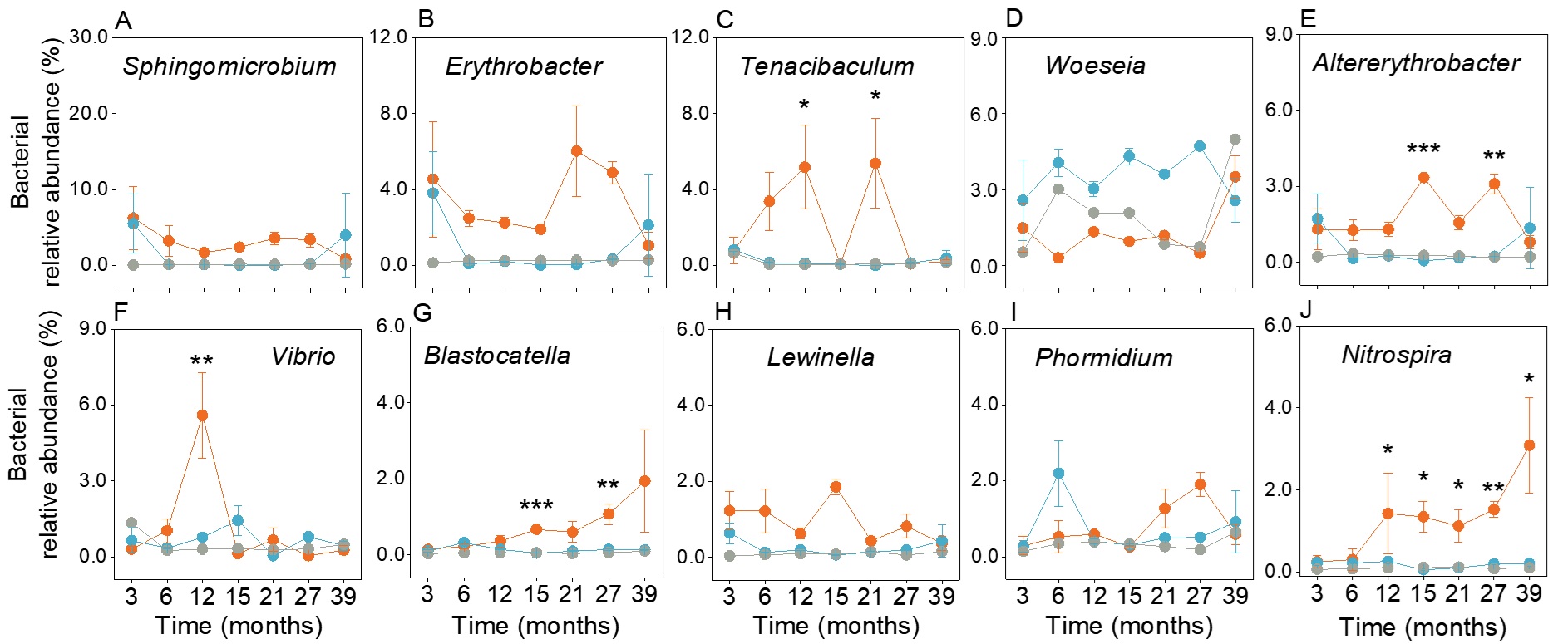


**Fig. S10**. **Time-course variations in** **the relative abundance of the top 10 bacterial taxa in the plastisphere, sediments, and seawater at the genus level.** For the plastisphere, significant differences between the 3-month-old samples and those from other time points were indicated by asterisks: * *P* < 0.05; ** *P* < 0.01; *** *P* < 0.001; for sediments and seawater, few time variations were found and thus not indicated.

**
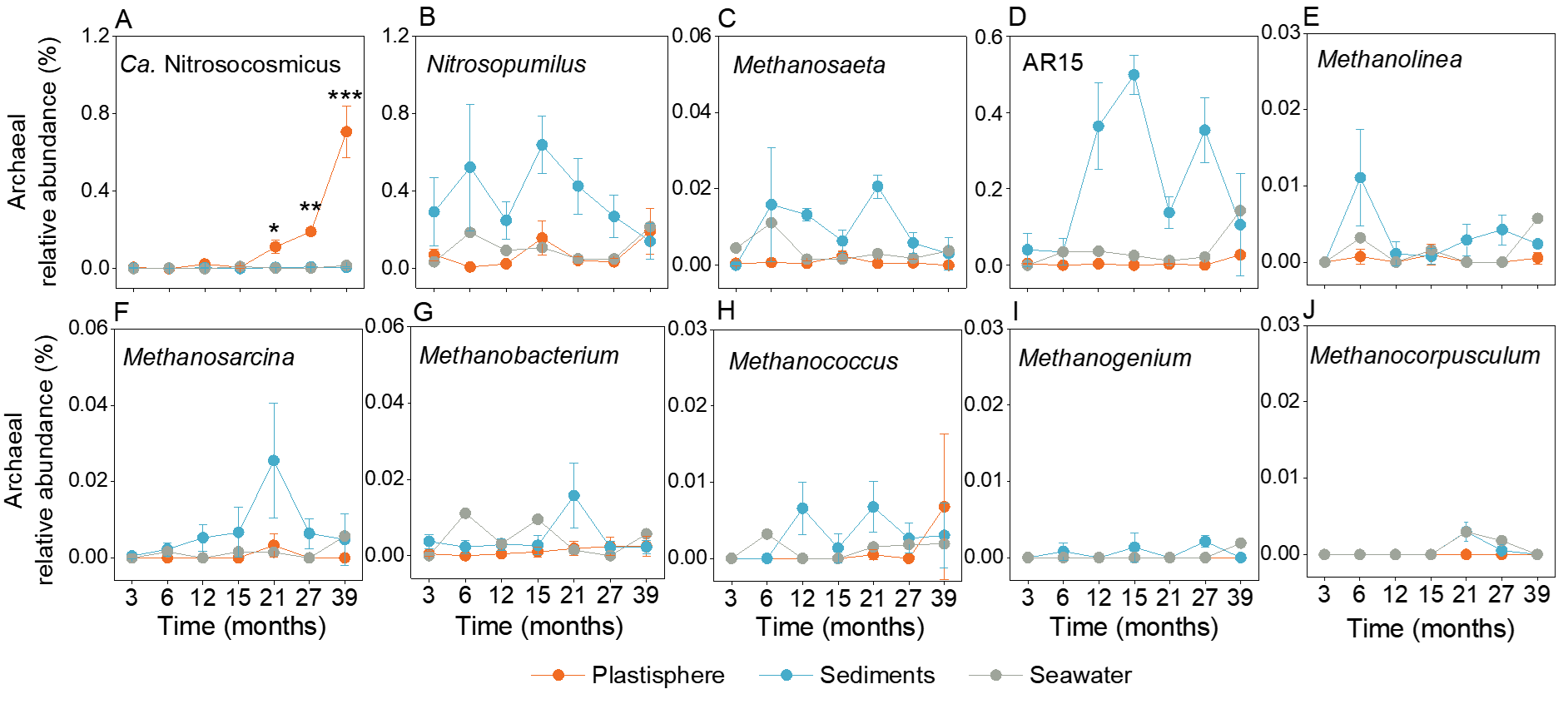
Fig. S11**. **Time-course variations in** **the relative abundance of the top 10** **archaeal taxa in the plastisphere, sediments, and seawater at the genus level.** For the plastisphere, significant differences between the 3-month-old samples and those from other time points were indicated by asterisks: * *P* < 0.05; ** *P* < 0.01; *** *P* < 0.001; for sediments and seawater, few time variations were found and thus not indicated. The archaeal 16S rRNA gene sequences were amplified with primers 515F and 806R.


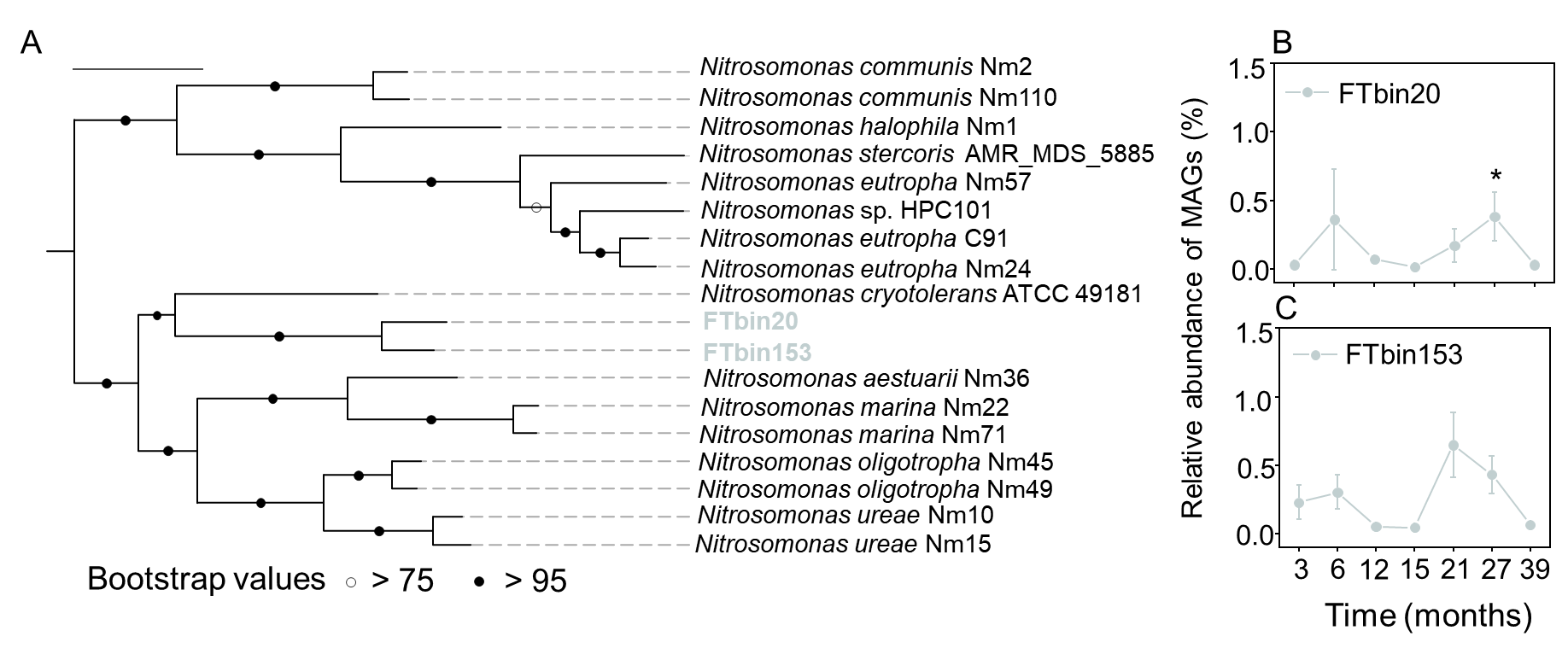


**Fig. S12.** **The relative abundance of the two retrieved ammonia oxidation MAGs.** (A) Phylogenomic analysis of the retrieved *Nitrosomonas* MAGs and reference genomes. *Nitrosospira* sp. NRS527 was used as the outgroup. The tree was calculated using the LG model from an alignment of 31376 amino acid positions derived from 369 genes. Bold blue represents the MAG obtained in this study; bootstrap support values ≥ 75% are indicated by gray circles, and ≥ 95% are indicated by black circles. Scale bars, 0.1 average amino acid substitutions per site. (B-C) Temporal dynamics of the relative abundance of two MAGs in the plastisphere. Significant differences between the 3-month-old samples and those from other time points were indicated by asterisks: * *P* < 0.05.

**
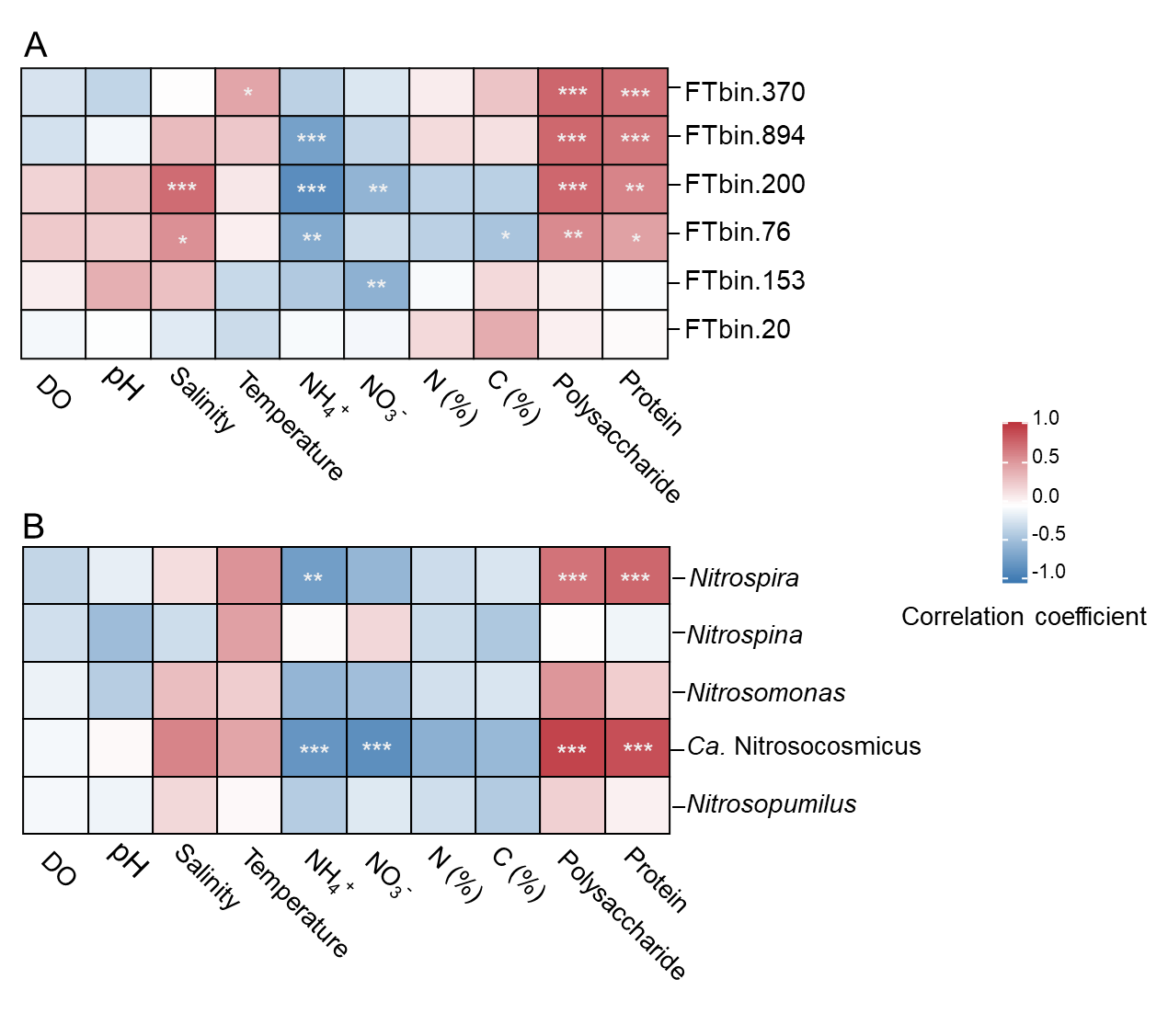
Fig. S13.** **Pearson correlations between the relative abundances of ammonia oxidizers and the concentrations of EPS components in the plastisphere and other physicochemical properties.** (A) The relative abundances of three ammonia oxidizers based on metagenomic binning. (B) The relative abundances of three ammonia oxidizers based on 16S rRNA gene amplicon sequencing analysis; * *P* < 0.05; ** *P* < 0.01; *** *P* < 0.001.


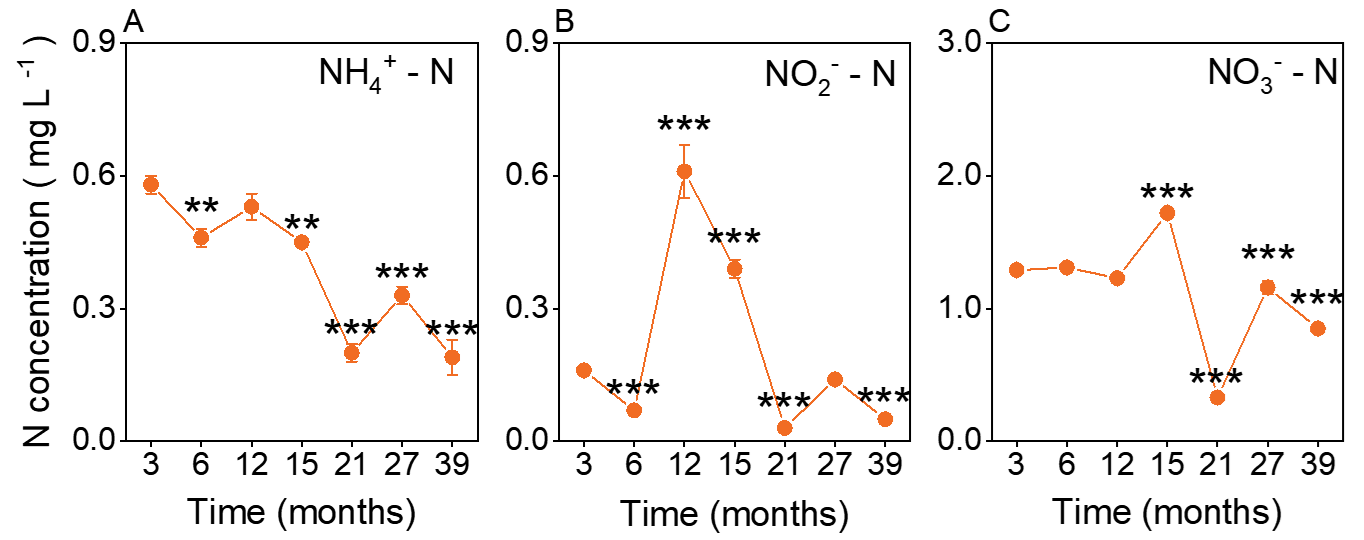


**Fig. S14**. **Time-course variations in nitrogenous compound concentrations in the seawater.** Error bars are the standard errors. Significant differences between the 3-month-old samples and those from other time points were indicated by asterisks: ** *P* < 0.01; *** *P* < 0.001.

**Table S1.** Information of 28 reference *amoA* genes of different ammonia oxidizers.

| **Microorganismm** | **Reference** | **Accession ID** |
| --- | --- | --- |
| AOA | *Nitrososphaera* sp. JG1 | JF748723 |
| AOA | *Nitrososphaera viennensis* | FR773159 |
| AOA | *Ca.* Nitrososphaera gargensis | EU281319 |
| AOA | WBM050405_45B4 | FJ227759 |
| AOA | A03 | KF286685 |
| AOA | *Ca.* Nitrosocosmicus hydrocola G61 | GCA_001870125.1 |
| AOA | *Ca.* Nitrosocosmicus sp. HNSD | MK396101 |
| AOA | *Ca.* Nitrosocosmicus sp. SS | MH024494 |
| AOB | 5 -12 | EF204978 |
| AOB | *Nitrosomonas oligotropha* Nm75 | KU747129 |
| AOB | *Nitrosomonas* sp. MAG_47 | GCA_021582795.1 |
| AOB | *Nitrosomonas* sp. AL212 | AF327918 |
| AOB | *Nitrosomonas marina* Nm22 | AJ388587 |
| AOB | St12-FL-14 | HQ330936 |
| AOB | St10-FL-25 | HQ330922 |
| AOB | *Ca.* Methylumidiphilus alinenensis | MH367014 |
| AOB | *Nitrosococcus oceani* ATCC 19707 | AF047705 |
| AOB | *Nitrosococcus halophilus* | AF272521 |
| CMX | KM1b-e | AJ868245 |
| CMX | Coma_*amoA*_37 | MF346972 |
| CMX | Coma_*amoA*_40 | MF346975 |
| CMX | Coma_*amoA*_35 | MF346970 |
| CMX | Coma_*amoA*_36 | MF346971 |
| CMX | *Ca.* Nitrospira nitrificans | GCA_001458775.1 |
| CMX | PS.N.2 | AB222896 |
| CMX | *Nitrospira inopinata* ENR4 | GCA_001458695.1 |
| CMX | *Ca.* Nitrospira nitrosa | GCA_001458735.1 |
| CMX | OA38 | FN394207 |

| **Microorganism** | **Reference** | **Accession ID** |
| --- | --- | --- |
| CMX | *Nitrospira* sp. CR1.3 | GCA_014055525.1 |
| CMX | *Nitrospira* sp. CG24E | GCA_002869895.2 |
| CMX | *Nitrospira* sp. RCB | GCA_005239475.1 |
| CMX | *Nitrospira* sp. RSF7 | GCA_005116825.1 |
| CMX | *Nitrospira* sp. RSF3 | GCA_005116835.1 |
| CMX | *Nitrospira* sp. ST-bin5 | GCA_002083555.1 |
| CMX | *Nitrospira* sp. RSF13 | GCA_005116865.1 |
| CMX | *Nitrospira* sp. CG24D | GCA_002869855.2 |
| CMX | *Nitrospira moscoviensis* | GCF_001273775.1 |
| CMX | *Nitrospira* sp. RCA | GCA_005239465.1 |
| CMX | *Nitrospira* sp. UBA2082 | GCA_002331335.1 |
| CMX | *Nitrospira* sp. ST-bin4 | GCA_002083565.1 |
| CMX | *Nitrospira* sp. SG-bin2 | GCA_002083405.1 |
| CMX | *Nitrospira* sp. UBA5698 | GCA_002420115.1 |
| CMX | *Nitrospira* sp. UW-LDO-01 | GCA_002254365.1 |
| CMX | *Nitrospira* sp. SG-bin1 | GCA_002083365.1 |
| CMX | *Nitrospira* sp. RSF12 | GCA_005116955.1 |
| CMX | *Nitrospira* sp. RSF9 | GCA_005116745.1 |
| CMX | *Nitrospira* sp. RSF1 | GCA_005116965.1 |
| CMX | *Nitrospira* sp. RSF5 | GCA_005116895.1 |
| CMX | *Nitrospira* sp. CG24B | GCA_002869845.2 |
| AOA | *Nitrososphaerales archaeon* TH1177 | GCA_014523515.1 |
| AOA | *Ca.* Nitrosocosmicus oleophilus | GCA_000802205.2 |
| AOA | *Ca.* Nitrosocosmicus sp. Dino_bin19 | GCA_009379865.1 |
| AOA | *Ca.* Nitrosocosmicus arcticus | GCF_007826885.1 |
| AOA | *Ca.* Nitrosocosmicus sp. SS | GCA_008389435.1 |
| AOA | *Ca.* Nitrosocosmicus franklandus | GCA_900696045.1 |
| AOA | *Ca.* Nitrosocosmicus sp. WS192 | GCA_013114705.1 |
| AOB | *Nitrosomonas communis* Nm2 | GCA_001007935.1 |
| AOB | *Nitrosomonas communis* Nm110 | GCA_900106545.1 |
| AOB | *Nitrosomonas halophila* Nm1 | GCA_900107165.1 |
| AOB | *Nitrosomonas stercoris* AMR_MDS_5885 | GCA_035444365.1 |
| AOB | *Nitrosomonas eutropha* Nm 57 | GCA_003201195.1 |
| AOB | *Nitrosomonas* sp. HPC101 | GCA_009833125.1 |
| AOB | *Nitrosomonas eutropha* C91 | GCA_000014765.1 |
| AOB | *Nitrosomonas eutropha* Nm24 | GCA_900116685.1 |
| AOB | *Nitrosomonas cryotolerans* ATCC 49181 | GCA_900143275.1 |
| AOB | *Nitrosomonas aestuarii* Nm36 | GCA_003046585.1 |
| AOB | *Nitrosomonas marina* Nm22 | GCA_900110145.1 |
| AOB | *Nitrosomonas marina* Nm71 | GCA_900111605.1 |
| AOB | *Nitrosomonas oligotropha* Nm45 | GCA_009833085.1 |
| AOB | *Nitrosomonas oligotropha* Nm49 | GCA_003050805.1 |
| AOB | *Nitrosomonas ureae* Nm10 | GCA_001455205.1 |
| AOB | *Nitrosomonas ureae* Nm15 | GCA_900206265.1 |

**Table S2.** Information on reference genomes of different ammonia oxidizers.

**Table S3.** Polymerase chain reaction (PCR) primer sets used in this study.

| **Target Organism** | **Gene** | **Primer Sequence**  **(5′-3′)** | **Reference** |
| --- | --- | --- | --- |
| AOA | Arch-*amoA* | Arch-*amoA*-F: STAATGGTCTGGCTTAGACG  Arch-*amoA*-R: GCGGCCATCCATCTGTATGT | Francis et al., 2005 [5] |
| AOB | *amoA* | *amoA*-F: GGACTTCACGCTGTATCTG  *amoA*-R: GTGCCTTCTACAACGATT | Chandran et al., 2008 [6] |
| CMX | Ntsp-*amoA* | Ntsp-*amoA*-162F: GGATTTCTGGNTSGATTGGA  Ntsp-*amoA*-359R: WAGTTNGACCACCASTACCA | Fowler et al., 2018 [7] |

**Table S4.** Characteristics of 6 MAGs reconstructed from the plastisphere metagenomes.

| Bin Id | Completeness  (%) | Contamination  (%) | Genome  size (M) | No. of  contigs | GC  (%) | N50  (bp) |
| --- | --- | --- | --- | --- | --- | --- |
| FTbin370 | 53.6 | 8.2 | 4.1 | 1611 | 55 | 3033 |
| FTbin894 | 66.3 | 9.8 | 2.7 | 531 | 55 | 5125 |
| FTbin76 | 93.8 | 2.1 | 2.9 | 760 | 29 | 4479 |
| FTbin200 | 97.1 | 2.9 | 2.8 | 356 | 34 | 12535 |
| FTbin153 | 98.9 | 5.5 | 3.3 | 276 | 45 | 21262 |
| FTbin20 | 94.7 | 2.5 | 3.4 | 517 | 45 | 9477 |

| **Query** | **Reference** | **Accession ID** | **AAI** |
| --- | --- | --- | --- |
| FTbin200 | *Ca.* Nitrosocosmicus sp. SMAG_U290 | GCA_036521355.1 | 65.0 |
| FTbin200 | *Ca.* Nitrosocosmicus sp. LS_FP_2_bin.144 | GCA_025934495.1 | 65.9 |
| FTbin200 | *Ca.* Nitrosocosmicus sp. Dino_bin19 | GCA_009379865.1 | 73.2 |
| FTbin200 | *Ca.* Nitrosocosmicus sp. SMAG_U8832 | GCA_035279165.1 | 73.0 |
| FTbin200 | *Ca.* Nitrosocosmicus sp. WA-bin7 | GCA_029194465.1 | 73.5 |
| FTbin200 | *Ca.* Nitrosocosmicus sp. 47_S61 | GCA_030452325.1 | 73.5 |
| FTbin200 | *Ca.* Nitrosocosmicus oleophilus MY3 | GCA_000802205.2 | 73.1 |
| FTbin200 | *Ca.* Nitrosocosmicus sp. SMAG_U881 | GCA_035699165.1 | 72.8 |
| FTbin200 | *Ca.* Nitrosocosmicus sp. SMAG_U8997 | GCA_035274085.1 | 73.0 |
| FTbin200 | *Ca.* Nitrosocosmicus sp. SS | GCA_009861625.1 | 72.8 |
| FTbin200 | *Ca.* Nitrosocosmicus arcticus Kfb | GCA_007826885.1 | 73.4 |
| FTbin200 | *Ca.* Nitrosocosmicus sp. 48_S62 | GCA_030452275.1 | 73.6 |
| FTbin200 | *Ca.* Nitrosocosmicus sp. SMAG_U8739 | GCA_035281065.1 | 73.9 |
| FTbin200 | *Ca.* Nitrosocosmicus sp. RBC_AOA2 | GCA_031316275.1 | 73.0 |
| FTbin200 | *Ca.* Nitrosocosmicus franklandus | GCA_900696045.1 | 79.1 |
| FTbin200 | *Ca.* Nitrosocosmicus sp. SMAG_U16420 | GCA_035454745.1 | 81.4 |
| FTbin200 | *Ca.* Nitrosocosmicus sp. SMAG_U1688 | GCA_036280415.1 | 82.1 |
| FTbin200 | *Ca.* Nitrosocosmicus sp. SMAG_U2428 | GCA_035765235.1 | 80.9 |
| FTbin200 | *Ca.* Nitrosocosmicus sp. LAS21 | GCA_036984325.1 | 84.1 |
| FTbin200 | *Ca.* Nitrosocosmicus sp. WS192 | GCA_013114705.1 | 84.4 |
| FTbin200 | *Ca.* Nitrosocosmicus sp. SMAG_U8918 | GCA_035276505.1 | 85.7 |
| FTbin200 | *Ca.* Nitrosocosmicus sp. RBC071 | GCA_902826075.1 | 88.8 |
| FTbin200 | *Ca.* Nitrosocosmicus hydrocola | GCA_001870125.1 | 88.8 |

**Table S5.** Average amino acid identities (AAI) between FTbin200 and other *Nitrosocosmicus* genomes.

**Table S6.** N_2_O yield (‰) of the canonical ammonia-oxidizers and comammox *Nitrospira* derived from pure culture and microcosm studies.

| **Organism** | **Experimental types** | **NH_4_^+^ additional** | **N_2_O yield (‰)** | **Reference** |
| --- | --- | --- | --- | --- |
| CMX | *N. inopinata* | 1.1 mM | 0.7 | Kit et al., 2019 [8] |
| AOA | soil microcosm studies | - | 0.5 | Hink et al., 2017 [9] |
| AOB |  | - | 1.0 |  |
| AOA |  | - | 0.4 | Hink et al., 2018 [10] |
| AOB |  | - | 0.9 |  |
| AOA | soil slurry studies | 1 mM | 0.6 | Giguere et al., 2017 [11] |
| AOB |  | 1 mM | 0.6 |  |
| AOA | soil slurry studies | 1 mM | 0.6 | Tzanakakis et al., 2019 [12] |
| AOB |  | 1 mM | 1.5 |  |
| AOA |  | 150 μg N g^-1^ | 0.7 |  |
| AOB | soil microcosm studies | 150 μg N g^-1^ | 2.0 | Tan et al., 2022 [13] |
| CMX |  | 150 μg N g^-1^ | 0.8 |  |
| AOA + CMX |  | - | 0.8 |  |
| AOA | soil microcosm studies | 150 μg N g^-1^ | 0.2-1.3 | Tan et al., 2024 [14] |
| AOB |  | 150 μg N g^-1^ | 1.3-4.6 |  |
| CMX |  | 150 μg N g^-1^ | 1.2-2.2 |  |
| AOA | soil microcosm studies | 50 μg N g^-1^ | 1.6 | Jiang et al., 2023 [15] |
| AOB |  | 50 μg N g^-1^ | 2.4 |  |
| CMX |  | 50 μg N g^-1^ | 0.2 |  |
| AOA | microcosm studies  (plastisphere) | - | 0.8 | this study |
| AOB |  | - | 1.2 |  |
| CMX |  | - | 0.3 |  |
| AOA | microcosm studies (sediments) | - | 0.8 |  |
| AOB |  | - | 1.7 |  |

**Table S7.** Cell-specific N_2_O production rates from pure culture and microcosm studies. It is assumed that each cell of AOA, AOB, and CMX contains 2.5, 1.0, and 1.0 *amoA* gene copies, respectively [16].

| **Organism** | **Experimental types** | **Cell-specific rate (amol cell^-1^ h^-1^)** | **Reference** |
| --- | --- | --- | --- |
| AOB | *N. europaea* ATCC 19718 | 58.0 | Shaw et al., 2006 [17] |
| AOB | *N. europaea* ATCC 25978 | 15.5 |  |
| AOB | *N. briensis* strain 128 | 4.2 |  |
| AOB | *N. multiformis* ATCC 25196 | 7.6 |  |
| AOB | *N. tenuis* strain NV12 | 2.0 |  |
| AOB | *Nitrosospira* sp. strain 40KI | 4.6 |  |
| AOB | *Nitrosospira* sp. strain En13 | 5.7 |  |
| AOB | *Nitrosospira* sp. strain NpAV | 3.9 |  |
| AOA | *N. viennensis* | 4.6 | Stieglmeier et al., 2014 [18] |
| CMX | *N. inopinata* | 0.5-0.8 | Tan et al., 2022 [13] |
| AOB | soil microcosm studies | 3.2-26.8^*^ | Tan et al., 2024 [14] |
| AOA |  | 0.1-0.6^*^ |  |
| CMX |  | 0.7-1.5^*^ |  |
| AOB | microcosm studies  (plastisphere) | 4.6 | this study |
| AOA |  | 0.3 |  |
| CMX |  | 0.03 |  |
| AOB | microcosm studies (sediments) | 5.3 | this study |
| AOA |  | 0.4 |  |

^*^estimated from Fig. 1 in Tan et al., 2023.

**References**

1. Stein LY. Insights into the physiology of ammonia-oxidizing microorganisms. *Curr Opin Chem Biol* 2019;**49**:9-15. https://doi.org/10.1016/j.cbpa.2018.09.003.

2. Han P, Wu DM, Sun DY et al. N_2_O and NO_y_ production by the comammox bacterium *Nitrospira* *inopinata* in comparison with canonical ammonia oxidizers. *Water Res* 2021;**190**:116728. https://doi.org/10.1016/j.watres.2020.116728.

3. Holland BJ, Hay JN. The thermal degradation of PET and analogous polyesters measured by thermal analysis–Fourier transform infrared spectroscopy. *Polymer* 2002;**43**:1835-47. https://doi.org/10.1016/S0032-3861(01)00775-3.

4. Fan C, Huang YZ, Lin JN et al. Microplastic constituent identification from admixtures by Fourier-transform infrared (FTIR) spectroscopy: the use of polyethylene terephthalate (PET), polyethylene (PE), polypropylene (PP), polyvinyl chloride (PVC) and nylon (NY) as the model constituents. *Environ Technol Inno* 2021; **23**:101798. https://doi.org/10.1016/j.eti.2021.101798.

5. Francis CA, Santoro AE, Oakley BB et al. Ubiquity and diversity of ammonia-oxidizing archaea in water columns and sediments of the ocean. *Proc Natl Acad Sci U S A* 2005;**102**: 14683-88. https://doi.org/10.1073/pnas.0506625102.

6. Chandran K, Love NG. Physiological state, growth mode, and oxidative stress play a role in Cd(II)-mediated inhibition of Nitrosomonas europaea 19718. *Appl Environ Microbiol* 2008;**74**:2447-53. https://doi.org/10.1128/AEM.01940-07.

7. Fowler SJ, Palomo A, Dechesne A et al. Comammox *Nitrospira* are abundant ammonia oxidizers in diverse groundwater‐fed rapid sand filter communities. *Environ Microbiol* 2018;**20**:1002-15. https://doi.org/10.1111/1462-2920.14033.

8. Kits KD, Jung MY, Vierheilig J et al. Low yield and abiotic origin of N_2_O formed by the complete nitrifier *Nitrospira inopinata*. *Nat Commun* 2019;**10**:1836. https://doi.org/10.1038/s41467-019-09790-x.

9. Hink L, Nicol GW, Prosser JI. Archaea produce lower yields of N_2_O than bacteria during aerobic ammonia oxidation in soil. *Environ Microbiol* 2017;**19**:4829-37. https://doi.org/10.1111/1462-2920.13282.

10. Hink L, Gubry-Rangin C, Nicol GW et al. The consequences of niche and physiological differentiation of archaeal and bacterial ammonia oxidisers for nitrous oxide emissions. *ISME J* 2018;**12**:1084-93. https://doi.org/10.1038/s41396-017-0025-5.

11. Giguere AT, Taylor AE, Suwa Y et al. Uncoupling of ammonia oxidation from nitrite oxidation: impact upon nitrous oxide production in non-cropped Oregon soils. *Soil Biol Biochem* 2017;**104**:30-8. https://doi.org/10.1016/j.soilbio.2016.10.011.

12. Tzanakakis VA, Taylor AE, Bakken LR et al. Relative activity of ammonia oxidizing archaea and bacteria determine nitrification-dependent N_2_O emissions in Oregon forest soils. *Soil Biol Biochem* 2019;**139**:107612. https://doi.org/10.1016/j.soilbio.2019.107612.

13. Tan C, Yin C, Li W et al. Comammox *Nitrospira* play a minor role in N_2_O emissions from an alkaline arable soil. *Soil Biol Biochem* 2022;**171**:108720. https://doi.org/10.1016/j.soilbio.2022.108720.

14. Tan C, Yin C, Zhang L et al. Deciphering the functional importance of comammox vs. canonical ammonia oxidisers in nitrification and N_2_O emissions in acidic agricultural soils. *Soil Biol Biochem* 2024:109415. https://doi.org/10.1016/j.soilbio.2024.109415.

15. Jiang L, Yu J, Wang S et al. Complete ammonia oxidization in agricultural soils: high ammonia fertilizer loss but low N_2_O production. *Glob Change Biol* 2023;**29**:1984-97. https://doi.org/10.1111/gcb.16586.

16. Huang L, Chakrabarti S, Cooper J, et al. Ammonia-oxidizing archaea are integral to nitrogen cycling in a highly fertile agricultural soil. *ISME Commun* 2021;**1**:19. https://doi.org/10.1038/s43705-021-00020-4.

17. Shaw LJ, Nicol GW, Smith Z et al. *Nitrosospira* spp. can produce nitrous oxide via a nitrifier denitrification pathway. *Environ Microbiol* 2006;**8**:214-22. https://doi.org/10.1111/j.1462-2920.2005.00882.x.

18. Stieglmeier M, Mooshammer M, Kitzler B et al. Aerobic nitrous oxide production through N-nitrosating hybrid formation in ammonia-oxidizing archaea. *ISME J* 2014;**8**:1135-46. https://doi.org/10.1038/ismej.2013.220.
